# Supplementary material for: Development of a modular patient-reported outcome and experience measure on patient needs and benefits in CLL (PBI-CLL)
Source: J Patient Rep Outcomes. 2025 Apr 29;9:45. doi: 10.1186/s41687-025-00882-5 (PMC12040787; doi:10.1186/s41687-025-00882-5)
Supplement: Supplementary file 1 — Supplementary Material 1: Supplement 1. Interview guideline content elicitation [file 41687_2025_882_MOESM1_ESM.docx]

Plain English summary

Chronic lymphocytic leukaemia (CLL) is a common type of leukaemia in adults. Until now, there is no questionnaire that measures the perspective of patients into therapy decisions. This study aimed to create a new electronic questionnaire to understand the needs and therapy goals of patients with CLL. We interviewed patients about their treatment goals and disease burden. Based on the results, we developed a new questionnaire, the Patient Benefit Index for CLL (PBI-CLL). This questionnaire has three parts to measure the outcomes of a treatment, the quality of the treatment process, and the general preferences of patients with CLL. Patients found the questionnaire useful and easy to use. The PBI-CLL is the first tool of its kind to assess patients' needs at the beginning of a treatment. It shows that patients have different needs and that it is important to have a tool for measuring treatment outcomes. This questionnaire can help doctors make personalized treatment decisions together with the patient and to evaluate if patients achieved their treatment goals. In this way, patients can be included into treatment decisions.
