# Supplementary material for: Development of a modular patient-reported outcome and experience measure on patient needs and benefits in CLL (PBI-CLL)
Source: J Patient Rep Outcomes. 2025 Apr 29;9:45. doi: 10.1186/s41687-025-00882-5 (PMC12040787; doi:10.1186/s41687-025-00882-5)
Supplement: Supplementary file 3 — Supplementary Material 3: Supplement 3. Interview guideline cognitive debriefing [file 41687_2025_882_MOESM3_ESM.docx]

Abstract

**Background**

Chronic lymphocytic leukaemia (CLL) is the most common form of leukaemia in adults in western countries. Asymptomatic patients are under clinical observation; when indication for treatment according to guidelines is met, treatment is initiated. When choosing from the numerous new treatment options, individual patient needs should be considered. To date, no instrument exists to capture these needs.

This project aimed to develop a modular electronic Patient-Reported Outcome Measure (ePROM) that assesses treatment goals and benefits in adult patients with CLL.

**Methodology**

The ePROM was developed based on the Patient Benefit Index (PBI) methodology which captures the importance of treatment goals as well as the achievement of these goals. The development considered the COSMIN guidelines and included semi-structured interviews with 28 patients with CLL and free-text questionnaires (n=15). Data were analysed via qualitative content analysis according to Kuckartz. The PBI-CLL was finalized through an expert consensus and cognitive debriefing interviews with 14 patients.

**Results**

The content elicitation showed that the individual treatment burden and treatment goals in CLL vary considerably between patients, underlining the heterogeneity of this patient group. Patients reported disease burden in their physical constitution as well as mental burden. Many patients’ main goal was to live normally and with the lowest impact possible through the CLL and its therapy. The PBI-CLL developed based on these data consists of three modules: therapy outcomes, process quality and relative treatment preferences. The cognitive debriefing interviews showed that patients find the instrument relevant, comprehensive, and comprehensible.

**Conclusions**

The PBI-CLL is the first instrument to assess patients' needs and benefits in CLL. The heterogeneity we found in patient needs and preferences underlines the importance of a modular instrument which measures treatment goals and benefits in a standardized way. The PBI-CLL shall support both patient-centred therapeutic decision making and treatment evaluation in clinical practice, as well as patient-centred benefit assessment in clinical and health care research. It should therefore be tested for its psychometric properties in a subsequent validation study.

**Keywords:** Patient-reported outcome measures, Chronic lymphocytic leukaemia, Disease burden, Patient needs, Treatment goals, Patient Benefit Index

Plain English summary

Chronic lymphocytic leukaemia (CLL) is a common type of leukaemia in adults. Until now, there is no questionnaire that measures the perspective of patients into therapy decisions. This study aimed to create a new electronic questionnaire to understand the needs and therapy goals of patients with CLL. We interviewed patients about their treatment goals and disease burden. Based on the results, we developed a new questionnaire, the Patient Benefit Index for CLL (PBI-CLL). This questionnaire has three parts to measure the outcomes of a treatment, the quality of the treatment process, and the general preferences of patients with CLL. Patients found the questionnaire useful and easy to use. The PBI-CLL is the first tool of its kind to assess patients' needs at the beginning of a treatment. It shows that patients have different needs and that it is important to have a tool for measuring treatment outcomes. This questionnaire can help doctors make personalized treatment decisions together with the patient and to evaluate if patients achieved their treatment goals. In this way, patients can be included into treatment decisions.

Introduction

Chronic lymphocytic leukaemia (CLL) is the most common type of leukaemia in adults in western countries with an incidence of 6 per 100,000 people in Germany; men are affected more often than women [1, 2] Studies show that health-related quality of life (HRQoL) is severely affected [3]. Mean age at diagnosis is 72-73 years, however, due to increasing routine blood testing, the disease is now often found in younger years [1, 4, 2]. CLL develops through a clonal expansion of mature CD5 positive B-cells within blood, bone marrow, and lymphoid tissues [2]. Patients diagnosed at an asymptomatic, early stage of are kept under clinical observation (watch and wait); once patients develop symptoms and/or show signs of bone marrow suppression, therapy is recommended [5, 6]. CLL affects people differently; some remain asymptomatic for a long time while others show quick progression and severe symptoms. For the decision on a specific therapy, different factors such as the cytogenetic and molecular genetic profile, comorbidities, comedication and treatment adherence need to be considered [2, 5]. Within the last decade, therapy options for CLL have developed rapidly [7]. With the emerging therapies such as small molecule inhibitors and monoclonal antibodies, chemoimmunotherapy is now rarely used; treatment duration varies between a fixed time period and continuous therapy [4, 8, 5].

Like the choice of therapy, treatment benefit is highly individual and does not only depend on effectiveness and treatment modalities but also on patient preferences and individual life situations [9]. Particularly regarding the increasing number of treatment options, assessment of individual patient preferences has become even more relevant for treatment planning [4, 10]. The patient-relevant benefit should be measured by a tool that reflects whether individual patient’s needs and expectations are fulfilled. As oncologists have been found to often misjudge patient goals, these can only be defined by the patients themselves [11]. Furthermore, studies found that patients and physicians have different priorities in the treatment of CLL [12, 9].

To include the patient’s perspective, patient-reported outcome measures (PROMs) are increasingly used in evaluating therapies [13]. To our knowledge, no instrument that measures treatment goals and benefit in CLL exists. Existing PROM measuring HRQoL in CLL such as the QLQ-CLL17 [14] and cancer generic instruments such as the EORTC QLQ-C30 [15] or FACT-G [16], measure impairment at one point in time but neither assess the personal benefit of the chosen therapy nor include patients’ individual preferences [17, 18].

To close this gap, this study aimed to develop a new PROM measuring patient needs and benefits in CLL. Its structure will be based on the established Patient Benefit Index (PBI) methodology which assesses the subjectively perceived treatment benefit through reaching therapy goals. The first part of the PBI, the Patient Needs Questionnaire, assesses the importance of different treatment benefits for the patient. The second part, the Patient Benefit Questionnaire, assesses the achievement of these goals. A weighted global score quantifies the overall patient-relevant benefit [19]. PBI versions have been developed and validated for various indications such as multiple sclerosis [20], psoriasis [21], allergic rhinitis [22], and peripheral artery occlusive disease [23] and the tool has been used successfully in health care and clinical research [24–27].

The PBI for CLL (PBI-CLL) shall measure the importance and achievement of therapy goals both regarding treatment outcome and treatment process. It will therefore include both a PROM and a patient-reported experience measure (PREM). It shall be applicable to all patient groups, including patients under clinical observation and patients who already receive treatment.

Methods

This non-interventional methods development study followed the COnsensus-based Standards for the selection of health Measurement INstruments (COSMIN) [28].

**Step 1: Concept elicitation**

To develop CLL-specific treatment goal items, we conducted a literature review, followed by qualitative interviews and a free-text survey (Figure 1).

*<<Please insert Figure 1 here>>*

**Literature review**

A non-systematic literature review provided an overview on existing instruments, related constructs, and tools. It also explored topics relevant regarding CLL patient's therapy goals. It was conducted in November 2022 on PubMed/Medline. Search terms included but were not limited to “chronic lymphocytic leukaemia”, “patient needs”, “patient preferences”, “treatment goals”, “instruments”, “evaluation”, “questionnaire”, “index”, “scale”, “tool”, “survey”. Two researchers (BH, student assistant) scanned titles and abstracts to find relevant publications and screened the reference lists of the relevant papers.

**Data collection**

Based on the literature research and previous PBI development studies [20-23; 33], an interview guideline for qualitative semi-structured interviews with patients (Supplement 1) was developed in consultation with oncologists; it was pilot-tested with a CLL patient. All interviews were conducted by a public health professional (BH) experienced in qualitative interviewing. Throughout the study, minor changes were made to the interview guideline after discussions with an experienced PRO researcher (CB). Patients and recruiting practices were compensated with 30 Euros per interview. The interviews took place from February until August 2023 via phone or Zoom, according to patient preference.

Additionally, a free-text survey questionnaire was completed by patients. It included questions regarding treatment goals and burden, similar to the interview guideline (Supplement 2).

**Data analysis**

All interviews were audio recorded, transcribed verbatim, and pseudonymized. All hand-written questionnaires were typed into Microsoft Word. Through qualitative content analysis according to Kuckartz [29], data was categorized in the software MAXQDA [30]. The analysis process included identifying all text passages relevant to the research question and then applying thematic codes on potential treatment goals, thereby iteratively developing a hierarchical coding system (e.g. with "waiting time" and "same doctor throughout" grouped under "administrative aspects"). Eight interviews and all 15 questionnaires were coded independently by two researchers (BH and JR), then comparing and consenting the results with feedback given by CB. The number of new codes per interview and per survey was documented with a saturation table; the recruitment of new study participants was ended when the saturation table showed no or only minor new codes reflecting patient needs or burden.

Step 2: Expert consensus

Based on the coding system, an item pool was generated. An expert panel consisting of haematologists, PROM experts and patient representatives developed a first draft of the PBI-CLL. Clinicians received a reimbursement of 100 Euros for their participation, patients 30 Euros.

**Step 3: Cognitive debriefing**

The PBI-CLL draft version was transferred into digital format (www.pbi-cll.de).

It was tested in cognitive debriefing interviews with CLL patients conducted via Zoom, unless the patient preferred to be interviewed by telephone, in which case they were sent the PBI-CLL on paper. The Think Aloud Method was used where patients are asked to fill in the questionnaire while expressing their thoughts verbally. Patients were asked about comprehensiveness (including completeness of all key concepts), comprehensibility (their ability to read and understand items, instructions, and response options) and relevance of each item (including appropriateness of recall period and response options). The interviews were conducted by a researcher experienced in qualitative interviewing (BH) using an interview guideline (Supplement 3). Minor changes of the interview guideline were made throughout the interview conduction, based on discussion between BH and CB.

All interviews were audio-recorded and transcribed verbatim. The data was analysed based on the content analysis by Kuckartz (see described above). After rounds of 4—5 interviews, the PBI-CLL was revised. Interviews were conducted until no further need for changes was found for two interviews in a row.

In all study phases, inclusion criteria were a diagnosis with CLL, being aged 18 years or older, and being fluent in the German language. Patients were recruited through patient advocacy groups, social media, oncological practices, and a specialized CLL consultation hour.

Results

**Patient characteristics**

A total of 57 patients were included. Patients were heterogeneous regarding gender, age, and disease status (Table 1).

**Table 1**. Patient characteristics of study participants

|  | **Interviews**  **(n = 28)** | **Survey**  **(n = 15)** | **Cognitive Debriefing**  **(n = 14)** |
| --- | --- | --- | --- |
|  | n (%) / Mean (Range) | | |
| Age (years) | 64.4 (44 – 86) | 65.3 (42 – 82) | 64.1 (52 – 83) |
| **Gender**  Female  Male | 11 (39%)  17 (61%) | 5 (33%)  10 (67%) | 8 (57%)  6 (43%) |
| **Highest school education**  Higher education (12 or 13 years)  Middle school (10 years)  General education (9 years)  Not stated | 16 (57%)  9 (32%)  3 (11%)  0 (0%) | 9 (60%)  2 (13%)  3 (20%)  1 (7%) | 9 (64%)  4 (29%)  1 (7%)  0 (0%) |
| **Living situation**  Alone  With spouse/partner/family | 6 (21%)  22 (79%) | 3 (20%)  12 (80%) | 2 (14%)  12 (86%) |
| Distance to oncologist (km) | 22.8 (1 – 220) | 25.0 (2 – 90) | 24.5 (2 – 120) |
| **Disease status**  Watch and wait  First-line  Second-line | 11 (39%)  11 (39%)  6 (21%) | 8 (53%)  6 (40%)  1 (7%) | 9 (64%)  3 (21%)  2 (14%) |
| Time since diagnosis (years) | 7.4 (0.3 – 28) | 3.9 (0.1 – 15) | 7.4 (1 – 15) |
| **Treatment setting**  Practice [niedergelassen]  Hospital outpatient clinic [Klinikambulanz]  University hospital  Not stated | 11 (39%)  3 (11%)  14 (50%)  0 (0%) | 3 (20%)  3 (20%)  8 (53%)  1 (7%) | 9 (64%)  1 (7%)  4 (29%)  0 (0%) |
| **Interview setting**  Zoom  Phone | 13 (46%)  15 (54%) |  | 7 (50%)  7 (50%) |

**Step 1: Concept elicitation**

The literature review confirmed that no tools to measure treatment goals and benefits in CLL exist. Therefore, the results of the literature review are not described in further detail; however, we used the results as a basis for prompts included in the interview guide, that is, topics to be addressed only if not reported spontaneously by the patient (e.g., application route; duration of recurrence-free period).

Through qualitative content analysis of the interviews and surveys, a category system with four categories and up to three hierarchy levels was developed: the first category covered outcome-related therapy goals and burden; the second covered provider-related goals and wishes (regarding doctors or other health workers); the third covered treatment-process-related goals and wishes. The fourth category covered general wishes regarding the therapy.

*Outcomes-related treatment goals and burden*

In this category, the physical impact of the disease and/or treatment was the most mentioned aspect. Many patients reported tiredness, exhaustion, and fatigue: “*I also notice that I get exhausted quickly, that I get tired quickly. […] You have to rest more quickly. You're no longer so productive*” (I05). The B symptoms of CLL (fever, night sweats, weight loss) were also mentioned by many patients: “*What I also had, which is one of the B symptoms, is night sweat. […] And in the beginning, I even had some fever episodes”* (I22). Other physical impacts were breathlessness, heart problems, sleep disruptions, and polyneuropathies. Many patients could not say with certainty whether symptoms were caused by the CLL itself or by the therapy. Some had been living with the disease for many years and believed that some physical impairments were increasing due to their age. However, there were also patients who reported no or only very light physical impairments due to the CLL.

Another category was the mental impact due to CLL. Some patients reported fears about their future: “*I'm always thinking about when it will get worse and how I will be able to cope with it*” (S02). Others felt a mental burden due to the CLL; this included both patients in the watch and wait phase and patients who received treatment: “*So far it’s only in my head. You think about the fact that you're carrying around a serious illness. And the watch and wait is of course also something that gives you cause for thought.*” (I26) “*The psychological burden is very great. Because you're constantly reminded of it. When I'm sitting at dinner with my wife and then something comes up, an infusion and then I have [to take] tablets, side effects, then I'm reminded of the CLL. Or when the fatigue becomes worse, I say: That damn illness*” (I19). Some patients said they do not feel any or only a very small impact on their mental health, particularly patients in the watch and wait phase: “*It's been going on for eight years now and I've been living very well with it for all eight years. And I hope it goes on like this for a few more years*” (I13).

Impacts on social life and relationships was mentioned by several patients. Some talked about reducing their social activities or avoiding crowds of people and the negative impact that the disease had on their social contacts: “*I don't go anywhere where lots of people gather. And if that happens, then I put a mask on. […] That restricts you. […] Because you're afraid that it will get bad*.” (I19). Several patients did not feel any impact on their social life: “*Overall, you can say that the social environment or social contacts do not change much*” (I24).

Further categories covered the influence of the disease on everyday life and leisure time: “*Holiday travels only possible to a limited extent. Limited sports activity*” (S04). Some patients reported having to reduce their work hours or volunteer work or that they “*had to stop working because of the illness*” (I07). Few patients felt financially impacted due to the CLL. Many hoped that through treatment, they could continue to live their life without any changes or impacts: “*My goal is of course to have as much normality as possible*” (I02).

*Provider-related treatment goals and wishes*

Patients mentioned the aspect of receiving information regarding their disease and the treatment. Some were hoping for information already when they first received their diagnosis, others also mentioned hoping to receive information regarding possible therapies early on: “*What if... I needed treatment? What if? How does that work? I am a person who needs a bit of a handrail, a guideline*” (I01). Most patients wanted extensive information regarding their disease in general and regarding the therapy once indication for treatment is given. Few said they did not want to receive a lot of information as they trusted their physician and would feel overwhelmed with information: “*But I'm not a doctor and he is known to be a good oncologist; if he thinks that would be good for me now, then I say: Yes, fine, then we'll do it*” (I09). Many patients also described finding information online, through forums or self-help groups.

Patients wished for a collaboration of the physicians involved in their treatment. Empathy and a trusting relationship to the doctor were important to many patients. Patients wanted to be involved in treatment decisions and wished to be taken seriously and have a relationship on an equal footing: *“I find that important, my opinion and my assessment are really accepted and taken seriously [by the new doctor]”* (I22). Lastly, patients wished for their physician to have extended knowledge regarding therapy options and to be well-informed and hoped for a positive attitude if they chose to get a second opinion.

*Treatment process-related goals and wishes*

Within this category, administrative aspects regarding the treatment were discussed. Some patients wished to always be treated by the same doctor, particularly in university hospitals. Others mentioned that they would appreciate appointments as rarely as possible and with short waiting times. Patients also mentioned they want good availability of the practice / clinic via phone or e-mail.

Furthermore, the circumstances regarding treatment application were important to some patients. Most patients preferred oral therapy over infusion, as it makes the treatment easier for them and they do not need to travel to their physician for treatment: “*I'd rather, because I can do it at home… I'd rather take tablets. […] Because of the [previous] infusions, I am completely bruised, I mean, my veins are*” (I14). While some patients wished for a break in their treatment if the clinical parameters improved, others did not mind their continuous therapy without any breaks: “*Of course it would be nice to pause [the treatment], but the treatment doesn’t restrict me at all*” (25).

*General wishes regarding the therapy*

This category included different general wishes regarding therapy, forming the basis for module 3 of the PBI-CLL which will provide a quick overview of what is important to the patient before a consultation. The preferences mentioned in this category were very heterogeneous. For example, some patients were willing to accept more severe side effects if this implied higher effectiveness of the treatment: “*Rather higher side effects at the beginning and a better cure. You can’t really speak of a cure for CLL*” (I10). Others wanted a treatment with as little side effects as possible: “*With the chemo it was very important that I had little side effects. […] So I was given a really mild mixture*“ (I03).

While some participants hoped to remain in the watch and wait phase for as long as possible, others preferred to receive treatment early on, hoping to increase the chances of remaining symptom-free: “*If there is a diagnosis, why don't you start relatively early a light […] or well-tolerated therapy? Why? Why do you wait until it has escalated?*” (I01).

Lastly, some patients preferred newer, innovative therapies while others wanted a tried and tested therapy. However, most patients mentioned that they want to avoid chemotherapy, mostly because they were scared about its side-effects: “*Without really knowing it, but you have images in your head or hearsay about what they can do to you or that they are quite stressful and so I have always avoided or still want to avoid that*” (I25).

For the full code system, see Supplement 4.

Based on the above-described categories, an item pool was developed by BH, CB and JR.

**Step 2: Expert consensus**

The item pool was examined by the expert panel in two rounds: in the first round, the item pool was discussed with a haemato-oncologist (JvT) and a patient with CLL; for organizational reasons, in the second round, the researchers included a further haemato-oncologist (MJV) in the discussion of the item pool.

A consensus was found on the content and structure of the questionnaire. The panel agreed on using the same response scale as in the original PBI version; for module three, the content of the questions as well as a bipolar format were agreed upon.

After the meetings, two researchers (BH, CB) made some additional small alterations (order of the items, phrasing, and grammar of items) and the expert panel confirmed the draft version of the questionnaire before the following study phase.

**Step 3: Cognitive debriefing**

Fourteen cognitive debriefing interviews, separated in three rounds, were conducted. In the first round, some patients mentioned unclarities regarding individual items. For example, multiple patients missed the symptom fatigue and did not think it was covered by the item on physical impairment; we added fatigue as a separate item. Additionally, some items were summarized, and the order of items was changed to increase comprehensibility. In the second round, only minor changes regarding the wording of individual items were made. In the third and last round, the final version of the questionnaire was tested with patients with no need for additional revisions found.

We found that most patients answered many needs items at the upper end of the scale; however, this cannot be quantified as interviewees' responses to the PBI-CLL were not documented.

Overall, patients found the questionnaire to be relevant and easy to understand: “*The questions were all understandable. They were very well phrased. And are basically understandable for everyone*” (CD13).

The final version of the questionnaire was uploaded to the website [www.pbi-cll.de](http://www.pbi-cll.de) and can be found in Supplement 5; for each treatment goal item, an example quote from the content elicitation interviews is given in Table 2. The original questionnaire was developed in German; for the process of translation into UK English, we followed the COSMIN guidelines [31]: the questionnaire was translated to English by two translators and back to German by two further translators; all translators had a mother tongue in the target or source language, respectively, were naïve on the construct measured by the PROM and worked independently from each other. In an online conference with both forward translators, the differences in the translations were discussed and a consensus was found. The preliminary version was checked by an additional translator and when finalized, discussed with the study team. The process of translation and all unclarities were documented in a separate document.

**Table 2: PBI-CLL treatment goal items (Patient Needs Questionnaire) and patient quotes**

| **Item No.** | **Treatment goal item (How important is it to you ...)** | **Patient quotes from content elicitation interview** | **Patient ID** |
| --- | --- | --- | --- |
|  | **Module 1: Importance of Treatment Outcomes** |  |  |
| 1 | ... that the therapy reduces or prevents fatigue (exhaustion and chronic tiredness)? | (...) the worst thing is the tiredness, which really affects you quite a bit. You sleep ten hours at night and can still sleep for two hours during the day. That's very intense. | I24 |
| 2 | ... that the therapy reduces or prevents the physical effects of CLL? | Regaining my stamina as much as possible. Running/ cycling/ strength (muscle building) sport in a group has always been important to me!  So it was this moment (...) that just made me happy. So free, the symptoms were suddenly gone and since then, as I said, everything has been fine, this moment when I could see that it was working and [the CLL was] regressing, the blood values got better and that was great, praise to the medicine, so to speak. | S01  I25 |
| 3 | ... that the therapy helps make your CLL less visible? | The symptom that the bones became thicker and thicker, that of course really bothered me, it no longer looked nice, I've occasionally been asked about it, and in that respect, it really bothered me | I25 |
| 4 | ... that the treatment is not time-consuming? | Well, I mean, back then with the chemotherapy, of course. You're tied down for six months. You have to go every four weeks. That was quite disruptive. Because depending on whether the blood count wasn't right, you had to extend [the treatment] by another week. | I15 |
| 5 | ... that the therapy disrupts your daily routine as little as possible? | Of course it's annoying that you always have the clock in your head because the medication has to be taken every day at the same time if possible, but I'd say that measured against what the medication does, I manage very well. Of course, I would prefer to take it once a year and then it would be just as good and, of course, it would be less effort, but that's a bit unrealistic. | I25 |
| 6 | … that the therapy you receive impairs your immune system as  little as possible? | During therapy, of course, you are simply weaker, more fragile and of course more vulnerable. You have to be even more careful. | I07 |
| 7 | … that the therapy is individually tailored to your comorbidities? | And people often forget that I have several diagnoses. And that's perhaps why my overall condition isn't so good. | I23 |
| 8 | … that the treatment helps you continue leading your life as before the diagnosis? | I personally don't define it in such a way that the goal is to reach 90 and then not really be able to live on it for the last 20 years, because for me, living is really a lot, working and sport and family, of course. That's actually my thing. And I just want to keep that going for as long as possible. That's actually the goal. | I02 |
| 9 | … that the treatment enables you to continue your working life with fewer limitations? | Well, it's a burden insofar as I've already mentioned that I'm self-employed. And of course, if something happens now, I wouldn't be able to take longer sick leave. Although, of course, I can take longer sick leave, but then I won't earn anything. (...) | I22 |
| 10 | … that the treatment enables you to enjoy a normal everyday life  and leisure activities? | I would like to, so I really enjoy travelling with my wife. Of course, I would like to do that again. For example, I don't dare to travel long distances at the moment. At the moment, I wouldn't travel further away from home than Europe so that I can get back within 24 hours or come back in case I get worse.  Can't do my everyday activities very well. Pain in my back, legs, head, neck | I24  S03 |
| 11 | … that the treatment enables you to enjoy a normal social life? | What I don't do now, I have to say quite clearly, I try to organise my holidays or free time in such a way that I meet as few people as possible. (...) People who have cancer and those around them know that they have cancer; that is very strange. They behave very differently to how they used to. They're afraid to talk to you, to talk about this illness at all, because they're afraid that they might embarrass themselves or that you might take offence or/ I don't know. But we've noticed that it's actually like a shield around you. | I19 |
| 12 | … that the treatment enables you to have a normal family life? | [My] children are more worried than I am. That's probably the case, you can't really prevent it either. And yes, cancer is actually very common in our family, I'd say. And of course the children end up worrying about how their father is doing and then it doesn’t help saying that I'm fine, because the family is just very worried. | I26 |
| 13 | … that the treatment makes you less anxious about a relapse? | And I still can't quite believe that it's really gone. That it won't come back. In other words, I'm thinking that it might come back somehow. And that's a bit of a worry, what will happen then? What will follow? Are there any options or are there only bad treatment options that restrict you more than they do now? | I21 |
| 14 | … that the treatment makes you less anxious about the future? | But of course it's not nice to know that you have two types of cancer, yes, which COULD get worse and worse. The theory is just bad. And that's why there are so many appointments. It's not always fun when you have to go there regularly every three months, every six months, whatever, and have to go for a check-up, isn't it? And, of course, you always expect the worst. You always expect the sentence: Oh [own name], now it's got a lot worse. And that's not nice, of course. You get phases here where you really ... see the dark side of it. | I23 |
| 15 | … that the treatment means you think less about your CLL? | Sometimes it gets more difficult, depending on where you come from/you always check the forums, what news are there? And then you read about the progress in the quiet phases or in the dark times that we've just had in the case, that's when it sometimes gets more difficult. Then you think: Oh gosh, that really shouldn’t have happened. | I01 |
| 16 | … that the treatment helps you feel less depressed? | Psychological is simply the strongest. Well, that's what I heard at the beginning: You'll live another 2 years, another 5 years. Then that was ... in the end more of a kind of death administration. Looking back, then. I stopped doing a lot of things because I thought there was no sense in it anyway. Why should I still do it? It was already limiting me mentally. [...] But ... when I look back on my life, it's rather disappointing. That I didn't do a lot of things because of the diagnosis. That's ... well, that's what burdens you the most. [...] | I03 |
| 17 | … that the treatment makes you less anxious about side effects? | So with the chemo it was very important that I had few side effects. You read so often about oral mucositis and fatigue, that the body is being killed. I didn't want any of that. (...) That was my goal. Gentle prolongation of life. And the leucocytes and all the blood values were back to normal after these 30 chemos. So I was out of the terminal stage, but in a super gentle way, I have to say. And I would have liked the same with the new drugs. I think the most important thing with CLL is to be gentle and maintain quality of life. I think that's incredibly important. | I03 |
| 18 | … that there is a minimal financial burden associated with CLL and the treatment? | It's extreme, the pressure I'm getting from the health insurance. So yes, I'm trying to take care of everything right now, but you can't really relax and when you're approached by 15 people from a health insurance, you have to deal with your worries alone/and then you have paperwork that you have to work through, which is anything but pleasant.  It's a collapse / I mean, I've never been anyone / I'm not a millionaire. But I've always worked normally and had my salary. And I could afford my two-bedroom flat, my car and all that. And now there's nothing left, right? (...) It's also a financial collapse, this whole medical history. It's also a burden/it's very stressful. | I18  I23 |
|  | **Module 2: Importance of process quality** |  |  |
| 1 | … to be given explanations and information about CLL, the course of this condition, and the therapeutic options available? | The important thing is how well you get it explained, what you have, what you can expect. | I07 |
| 2 | … to be given comprehensive information at the time of initial diagnosis? | I simply believe that the diagnosis in the initial consultation is the decisive key or decisive criterion, let's say, for how the patient deals with the issue in the first stage. It doesn't matter what it looks like in the individual case, but simply, let's say, to see the silver lining or the ray of sunshine on the horizon somewhere: Yes, this is what you have. You're at this stage. We do this and that. If you're at a different stage, we'll start therapy here and there. But you're in good hands with us. | I01 |
| 3 | … to be provided with brochures or written information when you receive your initial diagnosis? | So at the beginning I simply had no information at all. It was just bad. When I saw the second doctor at the clinic, for example, there were brochures. There were brochures from the German Cancer Aid, which I think would have been great if I'd gone there from the start or if the first doctor had given them to me. | I06 |
| 4 | … to be given an additional appointment soon after your initial diagnosis to provide you with further information, e.g., from trained practice staff? | And then I would like to be given a telephone number. That you can call again, say, three or four days later. Because it's/you're just sitting there in the doctor's room. You get that. And if, as I said, you're then also told: It's not bad. And thousands of people have it and so on. You don't want to hear that. And you don't believe it either. Because if I have cancer, I have cancer and cancer still has a bad aftertaste. (...) I'm always talking and doing and doing, so that you can just ask again afterwards. (...) That can also be a doctor's assistant. | I15 |
| 5 | … to receive information about future therapeutic options as early  as possible? | Well, to be relatively well informed, right? What are the options, what are the chances, what exactly are the treatment options if the time comes. In any case. I think the doctors always hold back a bit at first, if it doesn't come to a therapy, which is also normal so as not to fuel the fears. | I05 |
| 6 | … to receive information about support groups? | [...] I mean well, of course the doctors also do a lot to refer people to self-help groups. (…) I mean, with us, I'm lucky, it's also relatively easy in [city 1]. But for me, it's important when someone calls, when he has his diagnosis and before he takes Valium and all sorts of things, it's much better if you talk from one patient to the other | I15 |
| 7 | … to receive information about rehabilitation options or residential treatment at a medical spa? | [...] although there is no cure, it's important to me that I can perhaps do some kind of rehabilitation again, and I've already enquired about that. (...) just to leave everything here completely. And maybe go to rehab for two or three weeks. I'd like to do that too, maybe with my son. I still have to think about that, clarify whether that's possible. And yes, that's something that rounds off the therapy quite well for me. | I18 |
| 8 | … to be given information about the psychological support options, including for relatives if necessary? | I then also had psycho-oncological support. And that also helped me a lot. I thought that was very good. I also had to stop working because of the illness. And that psycho-oncologist was of course a great help, because I was very reluctant to stop working. And that was somehow very helpful. | I07 |
| 9 | … that your doctor is up-to-date with the latest research? | I want to be with a doctor who I know is up to date and can explain the therapy to me and also suggest alternatives and explain all the options with advantages and disadvantages so that I have the feeling that I am in really good hands. | I06 |
| 10 | … that the doctors involved in your treatment communicate with each other? | Exactly, so he's a haematologist and he's also directly in the clinic. So he has his practice in the clinic, so to speak, and of course the short distances and the short communication with various examinations and so on, that's relatively quick, yes, and of course that makes/is a big advantage. And the exchange with all his colleagues in oncology and so on. Of course, everything goes hand in hand. | I16 |
| 11 | … that the doctor treating you has a positive attitude towards obtaining a second opinion? | I have to say that I also think it's very, very positive that my haematologist took care of the appointment, that she forwarded all the documents to the university hospital. And, as I said, I got the call today within a week. That / or no, actually, I was there yesterday and the university hospital called me today. So that worked out really well, didn't it? I also think my doctor/ I also said here, if we were to start anything, that I would definitely like to get a second opinion. And then she says: No, no, definitely. That's not an issue. We'll do that too. | I05 |
| 12 | … that your doctor presents information clearly and in a way that is easy to understand? | For example, why don't doctors say, well I don't know if it's the same everywhere: if you have CLL, it's not curable. That word has never been used. But it's true. CLL IS NOT CURABLE. Why don't doctors tell you that? | I19 |
| 13 | … that your doctor talks to you as an equal? | I think a (…) doctor-patient relationship like that has to be somewhere on an equal footing. I would find it difficult (…) if I had the feeling that my doctors were saying: She doesn't understand what we're explaining to her anyway. That's why we only explain it to her in very broad terms. But I think that's also the case today / because everyone does research on the internet and has read so much. I think it's now the case that patients are given really comprehensive information. Which was also important to me at some point, but that has a bit to do with my personal history and my environment. | I07 |
| 14 | … that your doctor and healthcare staff are empathetic? | Of course empathy is always important. That's a very important topic. | I07 |
| 15 | … that your doctor takes time for you? | Yes, the important thing for me in any case was that the oncologist took his time. And he did. So there is such a / not that he says: You have CLL. Yes (incomprehensible), goodbye. No, he did: Do you have any questions? | I10 |
| 16 | … to have a good trust-based relationship with your doctor? | The important thing in treatment is to have a doctor you trust. And I always say that in our (self help) group, many people write: I have a good oncologist. Then I say to them: How do you know that your oncologist is good? You don't know anything about your illness, but you say your oncologist is good because he talks to you well, but you don't know if that's right. But I know with my doctors that I am/ that I am in good hands/ you have to feel that you are in good hands. You have to feel well looked after at the doctor's/ and I know exactly which doctors I go to. | I01 |
| 17 | … to always be treated by the same doctor? | Then I also went to the university hospital in [city 1], as I live in [city 1]. But I'm a statutory health insurance patient. So I realised that I would only have assistant (doctors) sitting in front of me. (…) They are at the cutting edge of research. Basically, I don't have a problem with that, but you sit in front of someone different every time and I thought it would be better to have a specific contact person for a disease. | I22 |
| 18 | … that waiting times at the practice or clinic are as short as possible? | You have long waiting times until you get an appointment. […] Always again, you wait four, five hours. But that is my time, that I don’t feel I have. Time is precious when you have CLL. | I03 |
| 19 | … that the practice or clinic is well organised? | So what I would like to see is the complete laboratory results, the blood count tests, if you could always get them straight away or if they were available in full. Of course, that's not really possible in terms of the procedure. (…) I would like to have all the data, also the attending physician to have all the data on the table. That you could also talk about it completely at the appointment, that would be something organisationally administrative that comes to mind. Something that could perhaps be improved. | I26 |
| 20 | … that the practice or clinic is easy to contact by email or  telephone? | That's great, of course. So if you can just write a quick email and get a quick reply. Or you can also call. That's great for me in terms of accessibility. And ... yes. Yes, that's actually the most important thing. | I02 |
| 21 | … how your medication is administered (e.g. tablets or drip)? | Of course, if you tolerate it well, it's always easier to take tablets ... than this infusion. Infusions are now a big problem for me because my veins are so bad. | I07 |
| 22 | … where the medication is administered (i.e. at home or in the hospital/medical practice)? | So if I/if the therapy was to start now, I would do it first, if I was to get an infusion or something else, as I have been told, I would definitely not want to do it at home. I will do it/ like the first therapy, I will go to hospital. I know that. Because I have a contact person there. And there I am ... on site. And I don't want to sit here at home and say to my husband: I'm not feeling well. (…) | I11 |
| 23 | … that if you take tablets, you only need to take them as  infrequently as possible? | Otherwise, if I could choose between two therapies, let's assume that one therapy would mean I would have to take tablets five times a day and the other only once, I would naturally tend towards the one with one tablet if they are equally safe. If, of course, the tablet therapy with the five tablets a day, which is what the research says, is much, responds much better, much longer in terms of experience, I would of course do that too. | I06 |
| 24 | … that the doctor will consider the possibility of pausing the therapy if your values have improved? | Of course, the main aim of any therapy is to remain treatment-free for as long as possible. Now that there is no cure, you naturally want to have a (treatment) break for many years.  I wouldn't take a break. So it's all right. You never know what will happen if you stop taking it for longer. (…) I'm a bit afraid that it will get worse again. I'm so glad that everything is going so well. | I06  I14 |
| 25 | … that you and your doctor reach decisions about the best treatment plan together? | Secondly, that I am included in the treatment. So, of course, many doctors already know that I, I mean it came, it is the case that I have a self-help group in [city 1] and of course they already knew that I argue differently, or rather, how should I describe it? I just want to be totally involved. I want to know everything. I want to know how long the treatment will last and so on. It is extremely important to me that I am properly involved as a partner. | I15 |
| 26 | … to receive detailed information about the treatment and its side effects at the start of treatment? | Yes, that I get properly informed about the, about the treatment options. And about the consequences of the various treatments and yes. Apart from that, that you can also talk about side effects. And that there are also solutions offered in that aspect. | I21 |

Discussion

This study aimed to develop an ePROM that assesses treatment needs and benefits in adult patients with CLL.

Throughout data collection, we included patients in the watch and wait phase as well as patients receiving treatment; the patient characteristics of participants also reflect the target population regarding age and gender [1, 2]. This supports the applicability across patient groups.

The data showed that patients have numerous therapy goals and report disease burden in different areas of their lives. While the physical impact of the disease and the treatment has a large effect on patients, also mental or psychological effects were reported by both watch and wait patients and patients undergoing treatment. Regarding the providers, patients mostly wished for empathic physicians, but found the expertise and knowledge of their provider even more important. Overall, most patients wanted normality in their lives: they hoped that the CLL or the therapy will not impact their lives and that a therapy will help to increase their life expectancy.

The cognitive debriefing interviews showed that participants found the PBI-CLL easy to use. They did not report difficulties completing the questionnaire in neither the electronic nor the paper-based version of the instrument (provided to patients who preferred to be interviewed over the phone). However, during the cognitive debriefing interviews, most patients answered a lot of needs items on the upper end of the scale, indicating a potential ceiling effect. Terwee and colleagues state that floor or ceiling effects may indicate limited content validity as patients in the lower and upper scores cannot be distinguished from each other [32]. However, most cognitive debriefing participants indicated that they would not prefer the response options to be expanded. As there were some patients who replied in the lower end of the Likert scale, we decided not to change the scale. Furthermore, the ceiling effect shows that patients found the goals to be of high importance and that the included items and the therapy goals are highly relevant for patients. In previous PBI developments, floor and ceiling effects were rather low [33], however, as this was part of the cognitive debriefing, our sample included only 14 patients compared to validation studies of other PBI versions. If a ceiling effect will be confirmed in a follow-up validation study, and assuming that it indicates that many outcomes are indeed of maximal importance for many patients, this will imply that determining an unweighted rather than a weighted global score will be sufficient to quantify patient benefit. In clinical practice, many high importance scores suggest that needs should be discussed broadly; few high importance scores suggest that discussion can be focused on specific areas. The validation study should also test the psychometric characteristics such as reliability, convergent validity, and responsiveness in a longitudinal design with a larger patient sample.

We expect that the PBI-CLL can be used in clinical practice as a basis for a discussion about therapy options and goals. It could provide the oncologist with a quick overview of what is important to patients; specific goals and wishes might be addressed more targeted. Therefore, the tool may save time during patient consultations. Additionally, it may increase satisfaction of patients who are included into treatment decisions and could indicate what is important to them. These assumptions need to be tested in subsequent studies.

The PBI-CLL data collection could be a challenge in clinical routine, as it will need to be integrated into existing data structures, with patients completing the questionnaire online before their visit or on a tablet while waiting. In some cases, it may be more feasible to use the paper version. Consideration should also be given to timing (e.g. first visit after diagnosis; before deciding on (new) treatment) and access to the data if there are multiple providers per patient. It is also important to avoid not discussing patients' responses in PBI-CLL, which can leave patients feeling unheard. In addition, incorporating the PBI-CLL assessment into clinic routines, deciding which modules to assess and when, and becoming familiar with its structure may require some initial effort, but this should be offset by the subsequent efficiency gains from structured assessment of patient needs and benefits. Once implemented, the PBI-CLL's unified Likert response scale should make it easy to quickly understand the data.

**Strengths and limitations**

A major strength is the involvement of patients in the study: already during the development of the study protocol, we involved self-help groups. During the progress of the study, we received a lot of positive feedback from patients and patient representatives in that they felt heard by being able to participate in the development.

In both the content elicitation and cognitive debriefing interviews, a small number of participants in the watch and wait phase reported having neither physical symptoms nor mental burden. These patients reported no therapy goals in the content elicitation and had difficulties filling in the PBI-CLL during the cognitive debriefing, as they found the therapy goals not to be relevant for them. This shows that, as expected, the goal achievement for module 1 (therapy outcomes) is not suited to patients in early watch and wait phase; they should only be asked to complete module 2 on process quality, and module 1 as soon as treatment is indicated. On the other hand, individual items that are not relevant to a patient do not pose a problem due to the "does not apply" response option.

Conclusion

This study provides an innovative electronic PROM that assesses treatment needs and benefits in patients with CLL, an indication for which only few PROMs exist. The instrument was developed according to the COSMIN standards and was seen as very positive by both patients and clinicians. It may help include patients into therapy decisions and provide a basis for a discussion of patient needs in patient-physician-consultations.

Abbreviations

CLL Chronic lymphocytic leukaemia

COSMIN COnsensus-based Standards for the selection of health Measurement INstruments

ePROM electronic Patient-Reported Outcome Measure

HRQoL Health-related quality of life

PBI Patient Benefit Index

PBI-CLL Patient Benefit Index for Chronic lymphocytic leukaemia

PREM Patient-reported experience measure

PRO Patient-reported outcome

PROMs Patient-reported outcome measures

References

1. Al-Sawaf O, Eichhorst B, Hallek M (2020) Chronische lymphatische Leukämie (Chronic lymphocytic leukemia). Internist (Berl) 61:277–287. https://doi.org/10.1007/s00108-019-00733-8

2. Hallek M, Shanafelt TD, Eichhorst B (2018) Chronic lymphocytic leukaemia. Lancet 391:1524–1537. https://doi.org/10.1016/S0140-6736(18)30422-7

3. Papathanasiou IV, Kelepouris K, Valari C et al. (2020) Depression, anxiety and stress among patients with hematological malignancies and the association with quality of life: a cross-sectional study. Med Pharm Rep 93:62–68. https://doi.org/10.15386/mpr-1502

4. Hallek M (2019) Chronic lymphocytic leukemia: 2020 update on diagnosis, risk stratification and treatment. Am J Hematol 94:1266–1287. https://doi.org/10.1002/ajh.25595

5. Tresckow J von, Eichhorst B, Bahlo J et al. (2019) The Treatment of Chronic Lymphatic Leukemia. Dtsch Arztebl Int 116:41–46. https://doi.org/10.3238/arztebl.2019.0041

6. Zhang S, Kipps TJ (2014) The pathogenesis of chronic lymphocytic leukemia. Annu Rev Pathol 9:103–118. https://doi.org/10.1146/annurev-pathol-020712-163955

7. Sharma S, Rai KR (2019) Chronic lymphocytic leukemia (CLL) treatment: So many choices, such great options. Cancer 125:1432–1440. https://doi.org/10.1002/cncr.31931

8. Parikh SA, Gale RP, Kay NE (2020) Chronic lymphocytic leukemia in 2020: a surfeit of riches? Leukemia 34:1979–1983. https://doi.org/10.1038/s41375-020-0852-7

9. Le H, Ryan K, Wahlstrom SK et al. (2021) Oncologist and Patient Preferences for Novel Agents in First-Line Treatment for Chronic Lymphocytic Leukemia: Commonalities and Disconnects. Patient Prefer Adherence 15:99–110. https://doi.org/10.2147/PPA.S289139

10. Le H, Pendergraft T, Wahlstrom SK et al. (2019) Understanding Clinician and Patient Preferences about Novel Agents in Chronic Lymphocytic Leukemia. Blood 134:4730. https://doi.org/10.1182/blood-2019-129606

11. Mesa RA, Miller CB, Thyne M et al. (2017) Differences in treatment goals and perception of symptom burden between patients with myeloproliferative neoplasms (MPNs) and hematologists/oncologists in the United States: Findings from the MPN Landmark survey. Cancer 123:449–458. https://doi.org/10.1002/cncr.30325

12. Laurenti L, Gaidano G, Mauro FR et al. (2022) What Are the Attributes Prioritized in the Choice of Therapy in Chronic Lymphocytic Leukemia? A Patient-physician Cross-matching Analysis of a Discrete Choice Experiment. Hemasphere 6:e771. https://doi.org/10.1097/HS9.0000000000000771

13. Unni E, Coles T, Lavallee DC et al. (2024) Patient adherence to patient-reported outcome measure (PROM) completion in clinical care: current understanding and future recommendations. Qual Life Res 33:281–290. https://doi.org/10.1007/s11136-023-03505-y

14. van de Poll-Franse L, Oerlemans S, Bredart A et al. (2018) International development of four EORTC disease-specific quality of life questionnaires for patients with Hodgkin lymphoma, high- and low-grade non-Hodgkin lymphoma and chronic lymphocytic leukaemia. Qual Life Res 27:333–345. https://doi.org/10.1007/s11136-017-1718-y

15. Aaronson NK, Ahmedzai S, Bergman B et al. (1993) The European Organization for Research and Treatment of Cancer QLQ-C30: a quality-of-life instrument for use in international clinical trials in oncology. J Natl Cancer Inst 85:365–376. https://doi.org/10.1093/jnci/85.5.365

16. Cella DF, Tulsky DS, Gray G et al. (1993) The Functional Assessment of Cancer Therapy scale: development and validation of the general measure. J Clin Oncol 11:570–579. https://doi.org/10.1200/JCO.1993.11.3.570

17. Bryant AL, Drier SW, Lee S et al. (2018) A systematic review of patient reported outcomes in phase II or III clinical trials of myelodysplastic syndromes and acute myeloid leukemia. Leuk Res 70:106–116. https://doi.org/10.1016/j.leukres.2018.06.006

18. Cannella L, Caocci G, Jacobs M et al. (2015) Health-related quality of life and symptom assessment in randomized controlled trials of patients with leukemia and myelodysplastic syndromes: What have we learned? Crit Rev Oncol Hematol 96:542–554. https://doi.org/10.1016/j.critrevonc.2015.07.012

19. Augustin M, Radtke MA, Zschocke I et al. (2009) The patient benefit index: a novel approach in patient-defined outcomes measurement for skin diseases. Arch Dermatol Res 301:561–571. https://doi.org/10.1007/s00403-009-0928-8

20. Beckmann H, Augustin M, Heesen C et al. (2019) Benefit evaluation in multiple sclerosis relapse treatment from the patients' perspective - Development and validation of a new questionnaire. Mult Scler Relat Disord 28:256–261. https://doi.org/10.1016/j.msard.2018.12.021

21. Feuerhahn J, Blome C, Radtke M et al. (2012) Validation of the patient benefit index for the assessment of patient-relevant benefit in the treatment of psoriasis. Arch Dermatol Res 304:433–441. https://doi.org/10.1007/s00403-012-1256-y

22. Franzke N, Schäfer I, Jost K et al. (2011) A new instrument for the assessment of patient-defined benefit in the treatment of allergic rhinitis. Allergy 66:665–670. https://doi.org/10.1111/j.1398-9995.2010.02515.x

23. Zander N, Demirel E-B, Augustin M et al. (2018) Development and validation of the Patient Benefit Index for peripheral arterial disease. Vasa 47:219–226. https://doi.org/10.1024/0301-1526/a000687

24. Costanzo A, Llamas-Velasco M, Fabbrocini G et al. (2023) Tildrakizumab improves high burden skin symptoms, impaired sleep and quality of life of moderate-to-severe plaque psoriasis patients in conditions close to clinical practice. J Eur Acad Dermatol Venereol 37:2004–2015. https://doi.org/10.1111/jdv.19229

25. Masuda K, Delozier AM, Kolodsick J et al. (2022) Improvements in itch and sleep following treatment with baricitinib in combination with topical corticosteroids are associated with better quality of life and productivity in adult patients with moderate-to-severe atopic dermatitis: a post hoc analysis from BREEZE-AD7 (Improvements in itch and sleep following treatment with baricitinib in combination with topical corticosteroids are associated with better quality of life and productivity in adult patients with moderate-to-severe atopic dermatitis: a post hoc analysis from BREEZE-AD7). Eur J Dermatol 32:237–243. https://doi.org/10.1684/ejd.2022.4242

26. Schräder NHB, Korte EWH, Duipmans JC et al. (2021) Identifying Epidermolysis Bullosa Patient Needs and Perceived Treatment Benefits: An Explorative Study Using the Patient Benefit Index. J Clin Med 10. https://doi.org/10.3390/jcm10245836

27. Thaçi D, Soliman AM, Eyerich K et al. (2021) Patient-reported outcomes with risankizumab versus fumaric acid esters in systemic therapy-naïve patients with moderate to severe plaque psoriasis: a phase 3 clinical trial. J Eur Acad Dermatol Venereol 35:1686–1691. https://doi.org/10.1111/jdv.17109

28. Terwee CB, Prinsen CAC, Chiarotto A et al. (2018) COSMIN methodology for evaluating the content validity of patient-reported outcome measures: a Delphi study. Qual Life Res 27:1159–1170. https://doi.org/10.1007/s11136-018-1829-0

29. Kuckartz U (2016) Qualitative Inhaltsanalyse: Methoden, Praxis, Computerunterstützung, 3., überarbeitete Auflage. Grundlagentexte Methoden. Beltz Juventa, Weinheim, Basel

30. VERBI GmbH MAXQDA 2022: computer software. VERBI-SOFTWARE 2021, Berlin, Germany

31. Mokkink, L. B., Prinsen, C., Patrick, D. L., Alonso, J., Bouter, L. M., De Vet, H. & Terwee, C. B. 2019 (2019, 1-32) COSMIN Study Design checklist for Patient-reported outcome measurement instruments. https://www.cosmin.nl/wp-content/uploads/COSMIN-study-designing-checklist_final.pdf. Accessed 20 Aug 2024

32. Terwee CB, Bot SDM, Boer MR de et al. (2007) Quality criteria were proposed for measurement properties of health status questionnaires. J Clin Epidemiol 60:34–42. https://doi.org/10.1016/j.jclinepi.2006.03.012

33. Blome C, Augustin M, Heyer K et al. (2014) Evaluation of patient-relevant outcomes of lymphedema and lipedema treatment: development and validation of a new benefit tool. Eur J Vasc Endovasc Surg 47:100–107. https://doi.org/10.1016/j.ejvs.2013.10.009

Figures

**Figure 1.** Flowchart Concept elicitation

Supporting Information

**Supplement 1.** Interview guideline content elicitation

**Supplement 2.** Written survey content elicitation

**Supplement 3.** Interview guideline cognitive debriefing

**Supplement 4.** Overview of the code system

**Supplement 5.** Questionnaire PBI-CLL, UK English version
