# Supplementary material for: Development of a modular patient-reported outcome and experience measure on patient needs and benefits in CLL (PBI-CLL)
Source: J Patient Rep Outcomes. 2025 Apr 29;9:45. doi: 10.1186/s41687-025-00882-5 (PMC12040787; doi:10.1186/s41687-025-00882-5)
Supplement: Supplementary file 5 — Supplementary Material 5: Supplement 5. Questionnaire PBI-CLL, UK English version [file 41687_2025_882_MOESM5_ESM.docx]

**PBI-CLL - Interview guideline**

**Thank you for** agreeing to take part in the study.

Brief **introduction**: My name is... I am at the (affiliation, position)...

With this survey, we would like to find out what **needs and goals** people with CLL have in relation to their disease. We address your **personal preferences, e.g. with** regard to a possible therapy, treatment by the oncologist and things that are important to you during the course of the disease.

Based on the results, we will create a **questionnaire** on treatment goals and treatment benefits that relates specifically to the treatment of CLL. This questionnaire can later be used by **doctors to** select a suitable treatment together with the patient, but also in **clinical studies** that compare the benefits of different treatments.

**NOTE: Ad hoc translation only for publication – Original guideline in German**

We look forward to hearing your **personal opinion** and getting to know your views; please do not mention any specific therapies. There is no right or wrong in anything you say; feel free to tell me openly what you would like to **tell** us about **the topic.**

I will always ask questions and draw our attention to certain aspects - but I will try to keep my own speaking time to a minimum so that we can **learn more about you and from you.**

I will audio-**record** our conversation and it will then be **written down verbatim**. We will remove personal information such as your name or places that you may mention in the conversation so that **no conclusions can be drawn** about you personally. Apart from this pseudonymized data (e.g. verbatim quotes to substantiate a statement), none of the information you share with me will be shared with anyone outside the study team.

The interview will last a maximum of **90 minutes** - if we are finished earlier, this is not a problem and is just as informative for us as a longer appointment. You can **stop** the interview at any time or take a **break** if you wish. You also have the option of **cancelling** your consent, in which case we will delete the recording.

Do you have any questions? Then I would **start** the **recording now**.

| **Questions** | **Remarks** |
| --- | --- |
| **Introductory questions** | |
| I would like to start by asking you to tell me **briefly** how you are **currently doing** with **CLL**. |  |
| Please tell me about how CLL has **affected** your life. |  |
| **Which therapies** have you already received?  *(Are you currently receiving therapy?)* |  |
| What **experiences** have you had with these therapies? | Not for W&W |
| In general, what aspects are **important to** you in the treatment of CLL? |  |
| **Specific subject areas** | |
| **Module 2** | |
| What is important to you in relation to the **therapy** and what are your goals? e*.g.*   - *Side effects / tolerability* - *Effectiveness* - *Innovation vs. tried and tested therapies* - *Stress caused by the treatment (e.g. psychological)* | Not for W&W |
| What is important to you with regard to the **type** and **external circumstances of the treatment**? What are your goals? e*.g.*  **NOTE: Ad hoc translation only for publication – Original guideline in German**   - *Frequency of treatment / role of treatment duration* - *Type of application* - *Compatibility* - *Approach to treatment* - *Treatment breaks* | Not for W&W |
| When you think about the entire treatment process - from your first visit to the doctor regarding CLL to the therapy: What is important to you about the **people treating you and the healthcare staff?** *e.g.*   - *Relationship with oncologist/healthcare staff* - *Provision of information on the disease and treatment* |  |
| What **benefits do** you expect from the treatment?  What do you hope the treatment will **improve** for you and in your life? | Not for W&W |
| What has **bothered** you about previous treatments? |  |
| **Module 1** | |
| It is often necessary to **weigh up** different treatment options.  Imagine you could choose between therapies **with more side effects** but often longer **freedom from recurrence** and therapies with **fewer side effects** but **a higher probability of subsequent recurrences**.  How do you feel about this? What is important to you? |  |
| How do you recognize that your treatment is or was successful?  *We have already talked about the benefits you expect from the treatment (list / summary of aspects).*  *If you had to rank these aspects in order of what is most important to you personally, what would it be?*  *(Examples: PFS, OS, QoL, tolerability, efficacy)* |  |
| **Module 3 & general questions** | |
| What impact do the CLL itself and the treatment have on your **quality of life**? | For W&W: only query the effects of CLL |
| To what extent do the CLL itself and the treatment affect you physically? Are there any important **physical** goals for you? |  |
| To what extent do CLL itself and the treatment affect you in your everyday life? Are there any important goals for you in the area of **everyday life**? |  |
| To what extent do CLL itself and the treatment affect you in your professional life? Do you have any important goals in your **professional life** or **voluntary work**? *(if relevant)* |  |
| To what extent do CLL itself and the treatment affect you in your free time? Are there any important goals for you in your **free time**?  **NOTE: Ad hoc translation only for publication – Original guideline in German** |  |
| To what extent do the CLL itself and the treatment affect your social relationships? Are there any important goals for you in the area of **social relationships**? |  |
| To what extent are the CLL itself and the treatment emotionally stressful? Are there important goals for you in the area of **psychological stress**? |  |
| **Conclusion** | |
| **Is there anything else** you think is important that we haven't mentioned yet? |  |

STOP RECORDING.

Finally, I would like to ask you for some **general information** about yourself.

- Age
- Gender
- Living situation: alone or with others (if yes, with whom?)
- Highest school-leaving qualification
- Duration of the disease
- Status of the disease: Watch & Wait / 1L /≥ 1st recurrence
- Treatment setting: outpatient practice (oncologist in private practice) / hospital outpatient clinic (not university hospital) / university hospital
- Distance to the treating oncologist (duration of journey)

**NOTE: Ad hoc translation only for publication – Original guideline in German**

Clarify at the end:

- Account details for expense allowance
- Note that payment of the expense allowance can take up to 8 weeks
- Information that participants will receive a brief summary of the study results at the end of the study
- Open questions

I would like to thank you very much for taking the **time to conduct** the interview with me and also for your **openness** during our conversation. These are very important insights that will help us in the development of the questionnaire.
