# Supplementary material for: Development of a modular patient-reported outcome and experience measure on patient needs and benefits in CLL (PBI-CLL)
Source: J Patient Rep Outcomes. 2025 Apr 29;9:45. doi: 10.1186/s41687-025-00882-5 (PMC12040787; doi:10.1186/s41687-025-00882-5)
Supplement: Supplementary file 6 — Supplementary Material 6 [file 41687_2025_882_MOESM6_ESM.docx]

**Development of a questionnaire on therapy goals and benefits for
patients with CLL**

How does CLL **affect** your life?

_________________________________________________________________________________________________________________________________________________________________________________________________________________________________

In general, what aspects are **important to** you in the **treatment of CLL**?

If you are not currently receiving treatment, what would be important to you?

____________________________________________________________________________________________________________________________________________________________________________________________________________________________________________________________________________________________________________

**NOTE: Ad hoc translation only for publication – Original questionnaire in German**

What is important to you in terms of the **type and circumstances of the treatment**?

(e.g. *the organizational circumstances of the treatment, the frequency of treatment, etc.*)

____________________________________________________________________________________________________________________________________________________________________________________________________________________________________________________________________________________________________________

When you think about the entire treatment process - from your first visit to the doctor about CLL to the therapy: What is important to you about the **people treating you and the healthcare staff?**

____________________________________________________________________________________________________________________________________________________________________________________________________________________________________________________________________________________________________________

What impact does the **treatment of CLL** have on your **quality of life**?

____________________________________________________________________________________________________________________________________________________________________________________________________________________________________________________________________________________________________________

To what extent does **CLL** affect you **physically**? What physical treatment goals do you have?

____________________________________________________________________________________________________________________________________________________________________________________________________________________________________________________________________________________________________________

**NOTE: Ad hoc translation only for publication – Original questionnaire in German**

To what extent does **CLL** affect you in your **everyday life**? What treatment goals do you have in relation to your everyday life?

____________________________________________________________________________________________________________________________________________________________________________________________________________________________________________________________________________________________________________

To what extent does **CLL** affect you in your **professional life** or **voluntary work**? What treatment goals do you have in relation to your job or voluntary work?

____________________________________________________________________________________________________________________________________________________________________________________________________________________________________________________________________________________________________________

To what extent does **CLL** affect you in your **free time**? What treatment goals do you have with regard to your leisure time?

____________________________________________________________________________________________________________________________________________________________________________________________________________________________________________________________________________________________________________

To what extent does **CLL** affect your **social relationships**? What treatment goals do you have in this area?

_________________________________________________________________________________________________________________________________________________________________________________________________________________________________

**NOTE: Ad hoc translation only for publication – Original questionnaire in German**

___________________________________________________________________________

To what extent does **CLL** affect you **emotionally**? What treatment goals do you have in this area?

____________________________________________________________________________________________________________________________________________________________________________________________________________________________________________________________________________________________________________

**General information:**

| Age: | _____ years |
| --- | --- |
| Gender: | female  male  diverse |
| Highest school-leaving qualification:  **NOTE: Ad hoc translation only for publication – Original questionnaire in German** | No school leaving certificate  Secondary school leaving certificate / primary school leaving certificate  Secondary school leaving certificate (intermediate school leaving certificate)  Advanced technical college entrance qualification  General higher education entrance qualification (Abitur)  Other school-leaving qualification, namely: ______________ |
| Living situation: | Alone  Together with ______________________ |
| Distance to the treating oncologist | Approx. ________ km |
| Disease exists since | _____ Year(s) n) |
| Current status of the disease: | Watch & Wait  First-line therapy (first therapy you receive for CLL)  ≥ 1st relapse (CLL has recurred after completion of the 1st therapy and must be treated again) |
| Place of treatment: | Outpatient practice / oncologist in private practice  Outpatient clinic (not university hospital)  University Hospital |

**Thank you very much for participating!**
