# Supplementary material for: Development of a modular patient-reported outcome and experience measure on patient needs and benefits in CLL (PBI-CLL)
Source: J Patient Rep Outcomes. 2025 Apr 29;9:45. doi: 10.1186/s41687-025-00882-5 (PMC12040787; doi:10.1186/s41687-025-00882-5)
Supplement: Supplementary file 7 — Supplementary Material 7 [file 41687_2025_882_MOESM7_ESM.docx]

**PBI-CLL - Cognitive Debriefing Interview Guide**

**Thank you for** agreeing to take part in the study.

Brief **introduction**: My name is... I am at the (affiliation, position)...

In the course of our study, we asked patients with CLL about their needs and goals. Based on the results of these surveys, we have created a **questionnaire on treatment goals and treatment benefits.** I would like to go through this questionnaire with you and see if it is **understandable** for you.

I will **record** our conversation and it will then be **written down verbatim**. We will remove personal information such as your name or places that you may mention in the conversation so that **no conclusions can be drawn** about you personally. Apart from this pseudonymized data (e.g. verbatim quotes to substantiate a statement), none of the information you share with me will be shared with anyone outside the study team.

**NOTE: Ad hoc translation only for publication – Original guideline in German**

The interview will last about **60 minutes** - if we finish sooner, this is not a problem and is just as informative for us as a longer appointment. You can **stop** the interview at any time or take a **break** if you wish. You also have the option of **cancelling** your consent, in which case we will delete the recording.

Do you have any questions? Then I would **start** the **recording now**.

| **Questions** | **COSMIN area** |
| --- | --- |
| Firstly, I would ask you to go through the questionnaire from start to finish and answer all the questions. How would you describe in your own words what this question says? *Do you have an example of what this could mean in concrete terms?*  In the meantime, please speak out loud any thoughts that come to mind. | Think Aloud |
| You hesitated with question XX - why was that? | Comprehensibility |
| Was there anything that you found difficult to understand in the wording of the questions or that you would improve / change? |  |
| Do you find the instructions at the beginning of the questionnaire understandable? What do you think could be improved or changed? |  |
| What do you think of the answer options? Do you find the answer options from "not at all" to "very" understandable/appropriate? Do the answer options match the questions?  **NOTE: Ad hoc translation only for publication – Original guideline in German** |  |
| *Does the reminder period describe an appropriate time?* |  |
| In your opinion, do the questions in the questionnaire cover all areas relating to treatment goals for CLL, or are you personally missing one or more aspects? | Completeness |
| *For questions that were ticked as not important*: Would you leave this question out of the questionnaire? | Relevance |
| *Are there any questions that you find unnecessary?* |  |
| Do you find the order of the items logical, or would you change the order? |  |
| Is there anything else you think is important that we haven't discussed yet? |  |

STOP RECORDING.

Finally, I would like to ask you for some **general information** about yourself.

- Age
- Gender
- Living situation: alone or with others (if yes, with whom?)
- Highest school-leaving qualification
- Duration of the disease
- Status of the disease: Watch & Wait / 1L /≥ 1st recurrence
- Treatment setting: outpatient practice (oncologist in private practice) / hospital outpatient clinic (not university hospital) / university hospital
- Distance to the treating oncologist (duration of journey)

Clarify at the end:

- Account details for expense allowance
- Note that payment of the expense allowance can take up to 8 weeks
- Information that participants will receive a brief summary of the study results at the end of the study
- Open questions

**NOTE: Ad hoc translation only for publication – Original guideline in German**

I would like to thank you very much for taking the **time to conduct** the interview with me and also for your **openness** during our conversation. These are very important insights that will help us in the development of the questionnaire.
