# Supplementary material for: Development of a modular patient-reported outcome and experience measure on patient needs and benefits in CLL (PBI-CLL)
Source: J Patient Rep Outcomes. 2025 Apr 29;9:45. doi: 10.1186/s41687-025-00882-5 (PMC12040787; doi:10.1186/s41687-025-00882-5)
Supplement: Supplementary file 8 — Supplementary Material 8 [file 41687_2025_882_MOESM8_ESM.docx]

| **Category level 1** | **Category level 2** | **Category level 3** |
| --- | --- | --- |
| Course of the disease* |  |  |
| Goals/burden | Time restrictions due to therapy |  |
|  | Everyday life and leisure |  |
|  | Restrictions due to COVID |  |
|  | Normality |  |
|  | Job/volunteering |  |
|  | Financial aspects |  |
|  | Physical | Inflammations |
|  |  | Difficulties with swallowing |
|  |  | Sleep disorders |
|  |  | Heartburn |
|  |  | Flavor impairments |
|  |  | Shortness of breath |
|  |  | Secondary diseases |
|  |  | Itching |
|  |  | Altered sleep patterns |
|  |  | Symptom triad (fever, weight loss, night sweat) |
|  |  | Externally visible effects |
|  |  | Bleeding (into the skin) |
|  |  | Cardiac arrhythmia/heart palpitations |
|  |  | Susceptibility to infections/weakened immune system |
|  |  | No physical effects |
|  |  | Cramps |
|  |  | Performance/fatigue/exhaustion |
|  |  | Other side effects (not specified) |
|  |  | Polyneuropathies |
|  |  | Pain |
|  |  | Swelling of the lymph nodes/spleen/liver |
|  |  | Nausea |
|  | Life expectancy |  |
|  | Mental/emotional | Fears about the future |
|  |  | Acceptance/distraction |
|  |  | Psychologically burdened |
|  | Social relationships | Talking about the disease |
|  |  | Avoidance of crowds/social activities |
|  |  | Strain on social relationships |
|  |  | No burden/goals |
| Goals/wishes: provider-related | Pleasant/friendly interactions |  |
|  | Cooperation between different physicians |  |
|  | Provision of information | General information |
|  |  | No further information desired |
|  |  | Information on support services/cures |
|  |  | Information when receiving initial diagnosis |
|  |  | Information on prospective therapy/therapy options |
|  |  | Information on therapy/side effects |
|  |  | Information on the disease |
|  |  | Communication in simple terms |
|  | Involvement in therapy decisions |  |
|  | Empathy |  |
|  | Expertise/correct therapy decision |  |
|  | Positive attitude |  |
|  | Psychological support |  |
|  | Factual presentation/honesty |  |
|  | Relationship at eye level/being taken seriously |  |
|  | Relationship of trust/"feeling in good hands" |  |
|  | Take time/cater to the patient |  |
|  | Second opinion |  |
| Goals/wishes: Treatment process | Administrative aspects | Cooperation within the practice |
|  |  | Same oncologist throughout treatment |
|  |  | Blood tests |
|  |  | Accessibility |
|  |  | Time of diagnosis |
|  |  | Appointments only if necessary |
|  |  | Waiting time |
|  |  | Availability of medication |
|  | Journey to treatment |  |
|  | Treatment breaks |  |
|  | Circumstances of application | Frequency of treatment |
|  |  | Type of application |
|  |  | Place of treatment |
|  |  | Other circumstances |
| Goals/wishes: Therapy | No deterioration |  |
|  | Symptom-free/treatment-free for as long as possible |  |
|  | Improvement of symptoms |  |
|  | Effectiveness |  |
|  | Improved performance/fitness |  |
|  | Aversion to chemotherapy |  |
|  | Acceptance of side effects |  |
|  | Innovation vs. tried and tested |  |
|  | Life extension |  |
|  | Palliative approach |  |
|  | General preferences |  |

*Icebreaker question during interviews
