# Supplementary material for: Development of a modular patient-reported outcome and experience measure on patient needs and benefits in CLL (PBI-CLL)
Source: J Patient Rep Outcomes. 2025 Apr 29;9:45. doi: 10.1186/s41687-025-00882-5 (PMC12040787; doi:10.1186/s41687-025-00882-5)
Supplement: Supplementary file 9 — Supplementary Material 9 [file 41687_2025_882_MOESM9_ESM.docx]

| **Module 1: Importance of Treatment Outcomes** |
| --- |

The following questions will help us find out how important the following goals are to you personally in the **current treatment** of your CLL. By "treatment", we mean both the **care** provided by your doctors as
well as any treatment with **medication**.

**not at all**

**somewhat**

**moderately**

**quite**

**very**

*does not apply to me*

**How important is it to you ...**

| 1 | ... that the therapy reduces or prevents fatigue (exhaustion and chronic tiredness)? | O | O | O | O | O |  | O |
| --- | --- | --- | --- | --- | --- | --- | --- | --- |
| 2 | ... that the therapy reduces or prevents the physical effects of CLL? | O | O | O | O | O |  | O |
| 3 | ... that the therapy helps make your CLL less visible? | O | O | O | O | O |  | O |
| 4 | ... that the treatment is not time-consuming? | O | O | O | O | O |  | O |
| 5 | ... that the therapy disrupts your daily routine as little as possible? | O | O | O | O | O |  | O |
| 6 | … that the therapy you receive impairs your immune system as  little as possible? | O | O | O | O | O |  | O |
| 7 | … that the therapy is individually tailored to your comorbidities? | O | O | O | O | O |  | O |
| 8 | … that the treatment helps you continue leading your life as before the diagnosis? | O | O | O | O | O |  | O |
| 9 | … that the treatment enables you to continue your working life with fewer limitations? | O | O | O | O | O |  | O |
| 10 | … that the treatment enables you to enjoy a normal everyday life  and leisure activities?  **NOTE: Preliminary translation only for submission – final version will be added once completed** | O | O | O | O | O |  | O |
| 11 | … that the treatment enables you to enjoy a normal social life? | O | O | O | O | O |  | O |
| 12 | … that the treatment enables you to have a normal family life? | O | O | O | O | O |  | O |
| 13 | … that the treatment makes you less anxious about a relapse? | O | O | O | O | O |  | O |
| 14 | … that the treatment makes you less anxious about the future? | O | O | O | O | O |  | O |
| 15 | … that the treatment means you think less about your CLL? | O | O | O | O | O |  | O |
| 16 | … that the treatment helps you feel less depressed? | O | O | O | O | O |  | O |
| 17 | … that the treatment makes you less anxious about side effects? | O | O | O | O | O |  | O |
| 18 | … that there is a minimal financial burden associated with CLL and the treatment? | O | O | O | O | O |  | O |

| **Module 2: Importance of process quality** |
| --- |

Using the following questions, we would like to find out how important the objectives of your **current** CLL **care** listed below are to you.

**not at all**

**somewhat**

**moderately**

**quite**

**very**

*does not apply to me*

**How important is it to you ...**

|  | | | | | | | | |
| --- | --- | --- | --- | --- | --- | --- | --- | --- |
| 1 | … to be given explanations and information about CLL, the course of this condition, and the therapeutic options available? | O | O | O | O | O |  | O |
| 2 | … to be given comprehensive information at the time of initial diagnosis? | O | O | O | O | O |  | O |
| 3 | … to be provided with brochures or written information when you receive your initial diagnosis?  **NOTE: Preliminary translation only for submission – final version will be added once completed** | O | O | O | O | O |  | O |
| 4 | … to be given an additional appointment soon after your initial diagnosis to provide you with further information, e.g., from trained practice staff? | O | O | O | O | O |  | O |
| 5 | … to receive information about future therapeutic options as early  as possible? | O | O | O | O | O |  | O |
| 6 | … to receive information about support groups? | O | O | O | O | O |  | O |
| 7 | … to receive information about rehabilitation options or residential treatment at a medical spa? | O | O | O | O | O |  | O |
| 8 | … to be given information about the psychological support options, including for relatives if necessary? | O | O | O | O | O |  | O |
| 9 | … that your doctor is up-to-date with the latest research? | O | O | O | O | O |  | O |
| 10 | … that the doctors involved in your treatment communicate with each other? | O | O | O | O | O |  | O |
| 11 | … that the doctor treating you has a positive attitude towards obtaining a second opinion? | O | O | O | O | O |  | O |
| 12 | … that your doctor presents information clearly and in a way that is easy to understand? | O | O | O | O | O |  | O |
| 13 | … that your doctor talks to you as an equal? | O | O | O | O | O |  | O |
| 14 | … that your doctor and healthcare staff are empathetic? | O | O | O | O | O |  | O |
| 15 | … that your doctor takes time for you? | O | O | O | O | O |  | O |
| 16 | … to have a good trust-based relationship with your doctor? | O | O | O | O | O |  | O |
| 17 | … to always be treated by the same doctor? | O | O | O | O | O |  | O |
| 18 | … that waiting times at the practice or clinic are as short as possible? | O | O | O | O | O |  | O |
| 19 | … that the practice or clinic is well organised? | O | O | O | O | O |  | O |
| 20 | … that the practice or clinic is easy to contact by email or  telephone? | O | O | O | O | O |  | O |

| **Module 2: Importance of process quality** |
| --- |

Using the following questions, we would like to find out how important the objectives of your **current** CLL **care** listed below are to you.

**not at all**

**somewhat**

**moderately**

**quite**

**very**

*does not apply to me*

**How important is it to you ...**

| 21 | … how your medication is administered (e.g. tablets or drip)?  **NOTE: Preliminary translation only for submission – final version will be added once completed** | O | O | O | O | O |  | O |
| --- | --- | --- | --- | --- | --- | --- | --- | --- |
| 22 | … where the medication is administered (i.e. at home or in the hospital/medical practice)? | O | O | O | O | O |  | O |
| 23 | … that if you take tablets, you only need to take them as  infrequently as possible? | O | O | O | O | O |  | O |
| 24 | … that the doctor will consider the possibility of pausing the therapy if your values have improved? | O | O | O | O | O |  | O |
| 25 | … that you and your doctor reach decisions about the best treatment plan together? | O | O | O | O | O |  | O |
| 26 | … to receive detailed information about the treatment and its side effects at the start of treatment? | O | O | O | O | O |  | O |

| **Module 3: Relative treatment preferences** |
| --- |

**For each of the following two statements, please indicate where you would place yourself on the scale.**

|  | **\|–––––––––\|–––––––––\|–––––––––\|–––––––––\|–––––––––\|–––––––––\|** | | | | | | |
| --- | --- | --- | --- | --- | --- | --- | --- |
| 1 | For a **high level of effectiveness**, I would accept severe side effects. |  |  |  |  |  | For a therapy with  **few side effects**, I would accept less effectiveness. |
|  |  |  |  |  |  |  |  |
|  | **\|–––––––––\|–––––––––\|–––––––––\|–––––––––\|–––––––––\|–––––––––\|** | | | | | | |
| 2 | If the level of effectiveness is the same, I would prefer a **proven** therapy that has been in use for a long time. |  |  |  |  |  | If they are equally effective, I would prefer a **new and innovative** therapy. |
|  |  | **NOTE: Preliminary translation only for submission – final version will be added once completed** |  |  |  |  |  |
|  | **\|–––––––––\|–––––––––\|–––––––––\|–––––––––\|–––––––––\|–––––––––\|** | | | | | | |
| 3 | When symptoms develop, I would prefer to **wait as long as possible** before starting therapy. |  |  |  |  |  | I would like to begin therapy **as early as possible**. |

| **Module 1: Achievement of therapy outcomes** |
| --- |

Please indicate the extent to which your treatment objectives **have been achieved** and therefore how beneficial the treatment has been for you.

**not at all**

**somewhat**

**moderately**

**quite**

**very**

*did not apply to me*

**The current therapy ...**

| 1 | … has reduced or prevented fatigue (exhaustion and chronic tiredness). | O | O | O | O | O |  | O |
| --- | --- | --- | --- | --- | --- | --- | --- | --- |
| 2 | … has reduced or prevented the physical effects of CLL. | O | O | O | O | O |  | O |
| 3 | … has helped make my CLL less visible. | O | O | O | O | O |  | O |
| 4 | … was not time-consuming. | O | O | O | O | O |  | O |
| 5 | … has not greatly disrupted my daily routine. | O | O | O | O | O |  | O |
| 6 | … has not greatly impaired my immune system. | O | O | O | O | O |  | O |
| 7 | … has been tailored to my comorbidities. | O | O | O | O | O |  | O |
| 8 | … has helped me continue leading my life as before the diagnosis. | O | O | O | O | O |  | O |
| 9 | … has enabled me to continue your working life with fewer limitations.  **NOTE: Preliminary translation only for submission – final version will be added once completed** | O | O | O | O | O |  | O |
| 10 | … has enabled me to enjoy a normal everyday life and leisure activities. | O | O | O | O | O |  | O |
| 11 | … has enabled me to enjoy a normal social life. | O | O | O | O | O |  | O |
| 12 | … has enabled me to have a normal family life. | O | O | O | O | O |  | O |
| 13 | … has made me less anxious about a relapse. | O | O | O | O | O |  | O |
| 14 | … has made me less anxious about the future. | O | O | O | O | O |  | O |
| 15 | … has helped me think less about my CLL. | O | O | O | O | O |  | O |
| 16 | … has helped me feel less depressed. | O | O | O | O | O |  | O |
| 17 | … made me less anxious about side effects. | O | O | O | O | O |  | O |
| 18 | … has ensured that there is a minimal financial burden associated with CLL and the treatment. | O | O | O | O | O |  | O |

| **Module 2: Achievement of process quality** |
| --- |

Please indicate the extent to which your objectives regarding the doctors and other people involved in your treatment **have been achieved** and therefore how beneficial the treatment has been for you.

**not at all**

**somewhat**

**moderately**

**quite**

**very**

*did not apply to me*

**During my CLL care thus far …**

| 1 | … I was given explanations and information about CLL, the course of this condition, and the therapeutic options available. | O | O | O | O | O |  | O |
| --- | --- | --- | --- | --- | --- | --- | --- | --- |
| 2 | … I was given comprehensive information at the time of initial diagnosis. | O | O | O | O | O |  | O |
| 3 | … I was provided with brochures or written information when I received my initial diagnosis. | O | O | O | O | O |  | O |
| 4 | … I was given an additional appointment soon after my initial diagnosis to provide me with further information, e.g., from trained practice staff. | O | O | O | O | O |  | O |
| 5 | … I received information about future therapeutic options as early as possible. | O | O | O | O | O |  | O |
| 6 | … I received information about support groups.  **NOTE: Preliminary translation only for submission – final version will be added once completed** | O | O | O | O | O |  | O |
| 7 | … I received information about rehabilitation options or residential treatment at a medical spa. | O | O | O | O | O |  | O |
| 8 | … I was given information about the psychological support options, including for relatives if necessary. | O | O | O | O | O |  | O |
| 9 | … my doctor was up-to-date with the latest research. | O | O | O | O | O |  | O |
| 10 | … the doctors involved in my treatment communicated with each other. | O | O | O | O | O |  | O |
| 11 | … the doctor treating me had a positive attitude towards obtaining a second opinion. | O | O | O | O | O |  | O |
| 12 | … my doctor presented information clearly and in a way that was easy to understand. | O | O | O | O | O |  | O |
| 13 | … my doctor talked to me as an equal. | O | O | O | O | O |  | O |
| 14 | … my doctor and healthcare staff were empathetic. | O | O | O | O | O |  | O |
| 15 | … my doctor took time for me. | O | O | O | O | O |  | O |
| 16 | … I had a good trust-based relationship with my doctor. | O | O | O | O | O |  | O |
| 17 | … I was always treated by the same doctor. | O | O | O | O | O |  | O |
| 18 | … waiting times at the practice or clinic were as short as possible. | O | O | O | O | O |  | O |
| 19 | … the practice or clinic was well organised. | O | O | O | O | O |  | O |
| 20 | … the practice or clinic was easy to contact by email or telephone. | O | O | O | O | O |  | O |

| **Module 2: Achievement of process quality** |
| --- |

Please indicate the extent to which your objectives regarding the doctors and other people involved in your treatment **have been achieved** and therefore how beneficial the treatment has been for you.

**not at all**

**somewhat**

**moderately**

**quite**

**very**

*did not apply to me*

**During my CLL care thus far …**

| 21 | … my preferences regarding how the medication is administered  (e.g. tablets or drip) were taken into account. | O | O | O | O | O |  | O |
| --- | --- | --- | --- | --- | --- | --- | --- | --- |
| 22 | … my preferences regarding where the medication is administered (i.e. at home or in the hospital/medical practice) were taken into account. | O | O | O | O | O |  | O |
| 23 | … I only needed to take the tablets infrequently. *(If you were not treated with tablets, please check "did not apply to me".)*  **NOTE: Preliminary translation only for submission – final version will be added once completed** | O | O | O | O | O |  | O |
| 24 | … the doctor considered the possibility of pausing the therapy once my values improved.  *(If there has been no improvement, please check "did not apply to me".)* | O | O | O | O | O |  | O |
| 25 | … my doctor and I reached decisions about the best treatment plan together. | O | O | O | O | O |  | O |
| 26 | … I received detailed information about the treatment and its side effects at the start of treatment. | O | O | O | O | O |  | O |
