# Supplementary material for: Development of a modular patient-reported outcome and experience measure on patient needs and benefits in CLL (PBI-CLL)
Source: J Patient Rep Outcomes. 2025 Apr 29;9:45. doi: 10.1186/s41687-025-00882-5 (PMC12040787; doi:10.1186/s41687-025-00882-5)
Supplement: Supplementary file 11 — Supplementary Material 11 [file 41687_2025_882_MOESM11_ESM.docx]

**Manuscript: "Development of a Modular Patient-Reported Outcome and Experience Measure on Patient Needs and Benefits in CLL (PBI-CLL)" (JPRO-D-24-00281)**

| Line | Comments | Reply |
| --- | --- | --- |
| 1 | **Reviewer #1:** |  |
| 2 | The manuscript describes the development of a modular electronic Patient-Reported Outcome Measure (ePROM) for patients with Chronic Lymphocytic Leukemia (CLL). The study is well-designed and addresses an important gap in the assessment of patient needs and benefits in CLL. The methodology is comprehensive and follows established guidelines. The results provide valuable insights into the heterogeneity of patient treatment goals and the importance of a modular instrument. The development of the PBI-CLL is highly relevant given the increasing complexity of treatment options for CLL and the need to consider patient preferences. By focusing on patient-reported outcomes and experiences, the instrument has the potential to improve patient-centered care and treatment decision-making. The study's findings regarding the heterogeneity of patient needs and the impact of CLL on various aspects of life (physical, mental, social) provide important insights for clinicians and researchers. This information can help in tailoring treatment plans and support services to individual patients.  However, there are areas that could be improved to enhance the clarity and impact of the study. | Thank you very much for carefully reviewing our study and giving valuable comments, which we believe have improved the manuscript. |
| 3 | Introduction  The introduction could provide a more detailed overview of the current challenges in CLL treatment and the limitations of existing patient-reported outcome measures. This would help to further emphasize the need for the PBI-CLL. A more in-depth discussion of the potential impact of patient-reported outcomes on treatment decisions and patient satisfaction could enhance the motivation for the study. | To further describe the need for the PBI-CLL, we added some more information on why PROMs, and specifically the PBI, are relevant and necessary:  “Like the choice of therapy, treatment benefit is highly individual and does not only depend on effectiveness and treatment modalities but also on patient preferences and individual life situations [9]. The perceived benefit does not only consist of an improvement in QoL after the intervention but also on individual preferences and perceived causal attribution to the therapy (Blome 2015). Particularly regarding the increasing number of treatment options, assessment of individual patient preferences has become even more relevant for treatment planning [4, 10]. The patient-relevant benefit should be measured by a tool that reflects whether individual patient’s needs and expectations are fulfilled. As oncologists have been found to often misjudge patient goals, these can only be defined by the patients themselves [11]. Furthermore, studies found that patients and physicians have different priorities in the treatment of CLL [12, 9]. According to previous studies, patients base their treatment decision on its effectiveness and also on compatibility with their lifestyle; the demonstrated superiority is here less important than the patient’s perceived effectiveness (Verghote et al. 2024). Additionally, priorities in the treatment do not only depend on the impact the disease has, but also on personal preferences which differ between patients (Blome 2016).” |
| 4 | Methods  In the description of the patient population, it would be beneficial to provide more information on the recruitment process, including the response rate and any potential biases. | Thank you for this comment. Unfortunately, we are not able to provide a response rate as it has not been systematically documented. We recruited patients through self-help groups and social media; here, it is not clear how many patients received the flyer. Furthermore, patients were recruited in a consultation hour. The clinician selected which patients met the inclusion criteria (for example, some patients had other types of leukaemia and therefore could not be included) and all eligible patients were approached by us; however, the response rate was not systematically documented – we added this information to the paper as well:  “Patients were recruited through patient advocacy groups, social media, oncological practices, and a specialised CLL consultation hour; the response rate was not systematically documented.” |
| 5 | Additionally, a comparison of the characteristics of the study participants with the general CLL population could help to assess the generalizability of the results. | Thanks for this suggestion. In the discussion, we compare the study participants with the general CLL population:  “Throughout data collection, we included patients in the watch and wait phase as well as patients receiving treatment; the patient characteristics of participants also reflect the target population regarding age and gender [1, 2]. This supports the applicability across patient groups.”  As this was a qualitative study, our aim was not to numerically map the ratios of patients but rather to ensure that all patient characteristics are broadly included and different perspectives can be shown, e.g. including patients from different age groups. |
| 6 | The description of the qualitative data analysis could be more detailed. For example, it would be useful to provide examples of the codes and categories developed and how they were refined through the iterative process. | We added more detail about analysis process and altered the manuscript to the following:  “The analysis process included identifying all text passages relevant to the research question and then applying thematic codes on potential treatment goals, thereby iteratively developing a hierarchical coding system. Using the interview guideline as a basis for categories, we categorised data into “goals/wishes” of different aspects: general (e.g. physical and mental/emotional), provider-related (e.g. provision of information, involvement in therapy decisions and positive attitude), related to the treatment process (e.g. administrative aspects, circumstances of application), and related to the therapy (e.g. acceptance of side effects and improved performance/fitness). These categories were then, if relevant, broken up into the third hierarchy level, e.g. with "waiting time" and "same doctor throughout" grouped under "administrative aspects". Eight interviews and all 15 questionnaires were coded independently by two researchers (BH and JR), then comparing and consenting the results with feedback given by CB. Throughout this process, all codes were reviewed and refined by all three researchers, e.g. by adding a third sub-category “fears about the future” to the category mental/emotional burden.”  Furthermore, an overview of all codes can be found in supplement 4. Additionally, table 2 provides an overview with example codes per item of the final questionnaire to point out what the basis for items was. |
| 7 | The translation process of the questionnaire could be described more clearly, including the specific steps taken to ensure equivalence between the German and English versions. | To clarify the different steps of the translation process, we changed the description of the translation to the following:  “The original questionnaire was developed in German: For the process of translation into UK English, we followed the COSMIN guidelines [31]: Firstly, the questionnaire was translated to English separately by two translators and then back to German by two further translators; all persons involved were professional translators from the translation agency lingoking, were mother tongue speakers in the target or source language, respectively, were naïve on the construct measured by the PROM and worked independently from each other. In an online conference with both forward translators and the questionnaire developers, the differences in the translations were discussed and a consensus was found to ensure comparability between the German and English version. The preliminary version was checked by an additional translator and when finalized, discussed with the study team. The process of translation was documented separately..” |
| 8 | Results  While the results are presented in a clear and organized manner, some of the tables could be made more reader-friendly. For example, in Table 2, the use of abbreviations and the large number of items could make it difficult for readers to quickly understand the content. Consider providing a more detailed explanation of the abbreviations and perhaps grouping the items in a more meaningful way. | Thank you for this positive feedback. As for the suggestion to change table 2, we made some changes to it: we added quotation marks to the quotes to clarify that these quotes are examples for how the items were formed. We also deleted some quotes when there were multiple quotes for one item, to ensure similarity between items. Further, we did not find any abbreviations other than the use of participant IDs – we added an explanation of the IDs in the bottom of the table. Overall, the table is structured similar to the final questionnaire. We also changed the description of the table to clarify its content:  “The final version of the questionnaire was uploaded to the website [www.pbi-cll.de](http://www.pbi-cll.de) and can be found in Supplement 5. Table 2 provides an example quote from the content elicitation interviews or the written survey for each treatment goal item to show how items were formed.“ |
| 9 | The discussion of the ceiling effect in the cognitive debriefing interviews could be more in-depth. It would be helpful to explore potential reasons for the effect and its implications for the interpretation of the results. Additionally, the decision not to change the response scale based on the feedback from the patients could be further justified. | Thank you for suggesting to discuss the potential ceiling effect in more detail. We think that the potential reason for the effect is that the therapy goals mentioned in the PBI-CLL are highly relevant for patients. We further explained why we decided not to change the scale and changed the section to the following:  “However, during the cognitive debriefing interviews, most patients answered a lot of needs items on the upper end of the scale, indicating a potential ceiling effect. Terwee and colleagues state that floor or ceiling effects may indicate limited content validity as patients in the lower and upper scores cannot be distinguished from each other [32]. However, most cognitive debriefing participants indicated that they would not prefer the response options to be expanded. For this reason, and because there were some patients who replied in the lower end of the Likert scale, we decided not to change the scale to ensure that replies on the lower end of the scale are also reflected in the questionnaire’s scores. Furthermore, the ceiling effect shows that most patients found the goals to be of high importance and that the included items and therefore the therapy goals are indeed highly relevant for patients. In previous PBI developments, floor and ceiling effects were rather low [33], however, as this effect was found in the cognitive debriefing, our sample included only 14 patients compared to validation studies of other PBI versions. If a ceiling effect will be confirmed in a follow-up validation study, and assuming that it indicates that many outcomes are indeed of maximal importance for many patients, this will imply that determining an unweighted rather than a weighted global score might be sufficient to quantify patient benefit. In clinical practice, many high-importance scores suggest that needs should be discussed broadly; few high-importance scores suggest that discussion can be focused on specific areas.” |
| 10 | Discussion  The discussion could further explore the potential applications of the PBI-CLL in clinical practice and research. For example, how could the instrument be used to compare different treatment modalities or to evaluate the effectiveness of patient-centered care interventions? | We agree that the comparison of treatments is an important point and added the following sentences:  “By using the PBI-CLL, oncologists will be able to assess better which information patients need and improve the quality of care as well as the patient satisfaction. These assumptions need to be tested in subsequent studies. The PBI-CLL can also be used to compare different therapies by comparing the mean scores for the therapies; this way, differences in the patient-reported benefit from interventions can be pointed out.” |
| 11 | The limitations section could be expanded to include a more comprehensive discussion of the potential biases and limitations of the study. This could include the impact of the small sample size in the cognitive debriefing interviews, the potential for recall bias in the qualitative | We agree that it is important to discuss potential biases and limitations of this study. The sample size of the cognitive debriefing interviews is in line with the COSMIN guidelines, which suggest a minimum of 7 interviews for the cognitive debriefing. To clarify, we added the following sentence:  “Due to the nature of the study which only included qualitative data, the results cannot be generalised; a quantitative validation study will be necessary to confirm psychometric characteristics.”  We also added the risk of recall bias to the limitations section:  “Conducting qualitative patient interviews also involves the risk of recall bias; however, including patients at different stages of their disease and/or therapy reduced and balanced this risk.” |
| 12 | **Reviewer #2:** |  |
| 13 | The authors have successfully addressed all comments and suggestions. | Thank you for this positive feedback. |
