# Supplementary material for: Development of a modular patient-reported outcome and experience measure on patient needs and benefits in CLL (PBI-CLL)
Source: J Patient Rep Outcomes. 2025 Apr 29;9:45. doi: 10.1186/s41687-025-00882-5 (PMC12040787; doi:10.1186/s41687-025-00882-5)
Supplement: Supplementary file 4 — Supplementary Material 4: Supplement 4. Overview of the code system [file 41687_2025_882_MOESM4_ESM.docx]

**Manuscript: "Development of a Modular Patient-Reported Outcome and Experience Measure on Patient Needs and Benefits in CLL (PBI-CLL)" (JPRO-D-24-00281)**

Dear Dr. Ruseckaite, dear reviewers,

Thank you very much for considering our manuscript for publication. Please find below our point-to-point reply to the reviewers’ comments.

Kind regards,

Beke Hester

| Line | Comments | Reply |
| --- | --- | --- |
| 1 | **Reviewer #1:** |  |
| 2 | Significance of the Research  Strengths: The manuscript addresses a significant gap in patient care for individuals with Chronic Lymphocytic Leukemia (CLL), particularly the absence of a dedicated tool for measuring patient needs and benefits in treatment selection and outcomes. The development of the Patient Benefit Index for CLL (PBI-CLL) is innovative and aligns with modern approaches in patient-centered care. The study emphasizes the heterogeneity of patient needs and presents a solution that can be applied in both clinical practice and research, supporting patient-centered therapeutic decisions. | Thank you very much for carefully reviewing our study and giving valuable comments, which we believe have improved the manuscript. |
| 3 | Weaknesses: While the study is novel, the paper would benefit from further contextualizing the practical implications of the PBI-CLL tool in routine clinical workflows. More explicit discussion on how its integration into diverse healthcare settings could be achieved would strengthen its relevance. | This is an important point. We have added the following paragraph to the discussion:  "The PBI-CLL data collection could be a challenge in clinical routine, as it will need to be integrated into existing data structures, with patients completing the questionnaire online before their visit or on a tablet while waiting. In some cases, it may be more feasible to use the paper version. Consideration should also be given to timing (e.g. first visit after diagnosis; before deciding on (new) treatment) and access to the data if there are multiple providers per patient. It is also important to avoid not discussing patients' responses in PBI-CLL, which can leave patients feeling unheard." |
| 4 | Originality  Strengths: This study is the first to develop a PROM (Patient-Reported Outcome Measure) specifically tailored for patients with CLL. The modular design, which accounts for therapy outcomes, process quality, and relative treatment preferences, reflects the variability of patient experiences, offering an original approach to addressing a highly individualized disease. | Thank you for this positive feedback. |
| 5 | Weaknesses:  Though original, the modular nature of the tool could introduce complexity. The manuscript should discuss potential challenges regarding the tool's usability in settings with limited resources or training in PROM usage. | We agree that it is important to discuss potential challenges in us of the PBI and have added the following to the discussion:  "In addition, incorporating the PBI-CLL assessment into clinic routines, deciding which modules to assess and when, and becoming familiar with its structure may require some initial effort, but this should be offset by the subsequent efficiency gains from structured assessment of patient needs and benefits. Once implemented, the PBI-CLL's unified Likert response scale should make it easy to quickly understand the data." |
| 6 | Methodology  Strengths: The methodology follows COSMIN guidelines and includes qualitative interviews with patients, an expert panel review, and cognitive debriefing interviews, ensuring a comprehensive and patient-centered approach. The inclusion of a diverse patient cohort (different disease stages and demographics) adds to the robustness of the data collected. | We appreciate this positive feedback. |
| 7 | Weaknesses:  The manuscript mentions a ceiling effect in the cognitive debriefing stage, where many patients selected the highest scores for importance. While the authors decided not to adjust the response scale, further clarification on the potential implications of this ceiling effect during future validation studies could enhance transparency. | Please note that the ceiling effects were only found for the needs questionnaire, not for the benefit questionnaire. We have specified this in the results section, where the respective sentence has been moved in line with the suggestion of reviewer 2:  "We found that most patients answered many needs items at the upper end of the scale; however, this cannot be quantified as interviewees' responses to the PBI-CLL were not documented."  "However, most cognitive debriefing participants indicated that they would not prefer the response options to be expanded."  We have addressed the implications of ceiling effects (which may or may confirmed in the larger follow-up study) in the discussion as follows:  "If a ceiling effect will be confirmed in a follow-up validation study, and assuming that it indicates that many outcomes are indeed of maximal importance for many patients, this will imply that determining an unweighted rather than a weighted global score will be sufficient to quantify patient benefit. In clinical practice, many high importance scores suggest that needs should be discussed broadly; few high importance scores suggest that discussion can be focused on specific areas." |
| 8 | Results and Data Analysis  Strengths: The results are well-presented, demonstrating the diversity of patient experiences and treatment goals. The authors successfully categorize findings into key areas (outcomes-related, provider-related, and process-related goals), which inform the structure of the PBI-CLL tool. | Thank you for these comments. |
| 9 | Weaknesses: While the qualitative content analysis is thorough, the manuscript could provide more detailed insights into how certain patient-reported experiences influenced the final instrument. For example, were there any significant differences in treatment goals between older and younger patients, or between those in different stages of the disease? | Thank you for this suggestion. Although the sample of study participants included patients with different experiences and characteristics (gender, age, education, living situation, disease status, time since diagnosis and treatment setting), the qualitative data analysis did not include a systematic investigation of differences between these patient subgroups. Instead, all potential treatment goals mentioned in the interviews were considered in the development of the PBI-CLL items, regardless of patient characteristics.  Due to the small sample size and the qualitative nature of most of the data on treatment goals, significance tests for group differences cannot be performed with our data set. This would need to be investigated in subsequent studies, such as the follow-up validation study that we are currently planning. |
| 10 | Discussion and Conclusion  Strengths: The discussion effectively highlights the practical utility of the PBI-CLL in enhancing patient-clinician communication and improving treatment planning by making patient preferences central to the decision-making process. The authors also recognize the need for further validation studies to confirm the psychometric properties of the tool. | Thank you for these comments. |
| 11 | Weaknesses: The authors briefly mention the role of psychometric validation as a next step but do not elaborate on the study design or parameters that will be tested. Providing more information on the expected timeline and scope for future validation efforts would strengthen the paper's conclusion and demonstrate a clear path forward. | We agree that an outline on the validation study would strengthen the paper. We are currently in the early planning process of the subsequent study, discussing the exact design, timelines, and also funding. Therefore, it is not yet possible to provide a reliable timetable. However, in the discussion, he have included more information on the design features that will be required in the validation study (briefly, given the word count limits):  "The validation study should also test the psychometric characteristics such as reliability, convergent validity, and responsiveness in a longitudinal design with a larger patient sample." |
| 12 | Clarity and Quality of Writing  Strengths: The manuscript is well-written, with clear, concise language that makes complex concepts accessible. The plain English summary is particularly effective in communicating the study's significance to a broader audience. | Thank you for the positive feedback. |
| 13 | Weaknesses: Some sections, such as the methodology, could benefit from more detail. For example, the process of thematic coding during the qualitative content analysis could be explained in more depth to enhance the reproducibility of the study. | Thank you for this suggestion; we have revised the Methods and particularly the section "Data analysis" for clarity. |
| 14 | Overall Recommendation  Major Revisions:  While the study presents important and original findings, the following areas require revision for improved clarity and impact -Expand the discussion on potential integration challenges of the PBI-CLL tool in routine clinical settings.  -Provide a more detailed explanation of the expected psychometric validation process.  -Address the ceiling effect more comprehensively, explaining how it may affect the tool's sensitivity in future use.  -Consider adding a supplementary table or appendix summarizing key patient quotes that influenced the final instrument design. | We understand that all but the last of these comments summarize comments you made above (please see our respective comments in lines 3, 5, 7, and 11 of this table).  Regarding the last comment, we added Table 2, PBI-CLL treatment goal items and patient quotes. In this process, some of the quotes provided in the main text have been moved to Table 2. |
| 15 | **Reviewer #2:** |  |
| 16 | The manuscript reports on a relevant topic, the development of a modular ePROM to assess treatment goals and benefits in adult patients with CLL.  To achieve this goal, they followed a very complex process supported by qualitative methodologies that the authors describe in detail, extensively describing the results and providing their conclusions. | Thank you for your thorough review and helpful comments. |
| 17 | However, I would like to provide some comments on some aspects of the manuscript that, in my opinion, could be improved.  Introduction  The introduction provides sufficient evidence to support the need to develop this new instrument. However, one can always be more concise in this section. | In line with your suggestion, we have revised the introduction to make it more concise (and to reduce the word count, which was needed after adding information in response to the reviewers' suggestions). |
| 18 | Method  This section has a few issues that need to be considered. In this section, the authors describe the complex process required to develop a new PROM based on the COSMIN guidelines.  They described several steps, 1) developing CLL-specific treatment items, which included literature review, preparation of interview guidelines with the oncologist, free-text survey completed by patients, data analysis; 2) generation of a set of items and a first draft of the instrument; 3) completion of the questionnaire followed by cognitive debriefing interviews.  The authors present data on the methods used for collection, the type of participants, the methodology used for analysis, including whether participants received monetary stipends for participating.  In the interest of being thorough in describing the methodology, the section may be somewhat confusing and difficult to follow for a reader unfamiliar with the procedure.  I would suggest being more concise by focusing on the most relevant aspects of the process, and accompanying the description in the text with a flow chart to clarify the different steps or phases, the participants in each step (type and number) and only a mention of the methodology or methodologies involved in that specific step in the chart, which will be described more extensively in the text.  In addition to making it easier to read, the chart could provide information on the three subsamples described in Table 1. | Thank you for this good idea – we have added the flowchart as Figure 1 and have revised the Methods section to make it more comprehensible and concise. |
| 19 | In Step 1, did the authors follow any guidelines for conducting the literature review? How did they process the content of the articles? In my opinion, there is a gap in the explanation of how the authors developed a qualitative interview (presumably semi-structured) based on the literature review. | Thank you for these comments.  The literature review did not follow specific guidelines. We there for added the term "non-systematic" to the description (Methods section):  "A non-systematic literature review provided an overview on already existing instruments, related constructs, and tools."  We have extended the development of the interview guideline (Methods section):  "Based on the literature research and previous PBI development studies [20-23; 33], an interview guideline for qualitative semi-structured interviews with patients (Supplement 1) was developed in consultation with oncologists; it was pilot-tested with a CLL patient.  We also provided more information on how the results of the literature review informed the interview guideline (Results section):  "To develop the content for the instrument, we used the data collected from interviews and free-text surveys. The literature review described in the methods confirmed our assumption that no tools to measure treatment goals and benefits in CLL. Therefore, the results of the literature review are not described in further detail; however, we used the results as a basis for prompts included in the interview guide, that is, topics to be addressed only if not reported spontaneously by the patient (e.g., application route; duration of recurrence-free period)."  The interviews were indeed semi-structured, as indicated in abstract; we have now added this information to the Methods section, too, as cited above. |
| 20 | Please clarify which interviews and questionnaires were administered in person, which via zoom or telephone, and why they define the instrument as ePROM when no electronic platform is described and the administration mode included online interviews via zoom or telephone and paper and pencil forms. | None of the concept elicitation or debriefing interviews were administered in person: "The interviews took place from February until August 2023 via phone or zoom." (Methods section).  We have added to the Methods section how it was decided whether phone or zoom interviews were conducted:  "The interviews took place from February until August 2023 via phone or Zoom, according to patient preference." [in the section describing the methods of concept elicitation interviews]  "It was tested in cognitive debriefing interviews with CLL patients conducted via Zoom, unless the patient preferred to be interviewed by telephone, in which case they were sent the PBI-CLL on paper." [in the section describing the methods of cognitive debriefing interviews]  Information on how many content elicitation interviews were conducted via zoom (n=13) and how many were conducted over the phone (n=15) can be found in Table 1. This table also reports that of the cognitive debriefing interviews, n=7 each were conducted via zoom and over the phone.  The ePROM version of the PBI-CLL was used in the Zoom interviews, for which it was implemented on a website: "The PBI-CLL draft version was transferred into digital format (www.pbi-cll.de)." (Methods section) |
| 21 | Results  The results are extensively and clearly reported based on qualitative data. | Thank you for this positive comment. |
| 22 | Discussion  The authors begin the discussion with a set of assumptions about the use of the instrument that need to be confirmed in future studies, for example, "The instrument can be used in daily clinical routine…" or "It provides the oncologist with a quick overview…", "…the tool can save time". These statements could be presented at the end of the discussion, such as, "it is expected that the instrument can be used in daily clinical practice or provide the oncologist with…", which will be questions for future research. | We agree that these are assumptions that need to be tested in future research. We have therefore shortened and revised the first paragraph to indicate that these potential benefits are assumptions. We have also moved this section to the end of the discussion, as suggested.  "We expect that the PBI-CLL can be used in clinical practice as a basis for a discussion about therapy options and goals. It could provide the oncologist with a quick overview of what is important to patients; specific goals and wishes might be addressed more targeted. Therefore, the tool may save time during patient consultations. Additionally, it may increase satisfaction of patients who are included into treatment decisions and could indicate what is important to them." |
| 23 | The authors comment on quantitative data referring to floor and ceiling effects coming from the responses to the questionnaire prior to the cognitive debriefing interview that were not presented in Results. | Thanks for this suggestion. We have revised the statement and moved it to the results section:  "We found that most patients answered many needs items at the upper end of the scale; however, this cannot be quantified as interviewees' responses to the PBI-CLL were not documented." |
| 24 | At the end of the section, the authors identify an important limitation of the instrument in that at least part of it is not suitable for patients in the early stages of the disease. In my opinion, they should expand on this aspect. | We have expanded on this aspect as follows:  "In both the content elicitation and cognitive debriefing interviews, a small number of participants in the watch and wait phase reported having neither physical symptoms nor mental burden. These patients reported no therapy goals in the content elicitation and had difficulties filling in the PBI-CLL during the cognitive debriefing, as they found the therapy goals not to be relevant for them. This shows that, as expected, the goal achievement for module 1 (therapy outcomes) is not suited to patients in early watch and wait phase; they should only be asked to complete module 2 on process quality, and module 1 as soon as treatment is indicated. On the other hand, individual items that are not relevant to a patient do not pose a problem due to the "does not apply" response option." |
| 25 | **Reviewer #3** |  |
| 26 | Overall, your manuscript is very well-written on a fascinating instrument that is of the utmost importance to patients and practitioners alike. Your description of the methodology used was clear, and you included relevant limitations to inform future iterations of the study. I also appreciated the use of current literature to support your work. I honestly do not have any constructive criticism to offer (which is rare for me when reviewing manuscripts), so thank you for your important contribution to the literature and to the field! | We appreciate this positive feedback. |
| 27 | **Further remarks** | Please note that as we have added content in response to reviewers' suggestions, we have shortened the text in other sections in order to stay within the word limit. All changes are highlighted, |
| 28 |  | Supplement 5: The preliminary translation of the PBI-CLL has been replaced by the final, linguistically validated UK English version. |
